# Assessing the application of landmark-free morphometrics to macroevolutionary analyses

**DOI:** 10.1186/s12862-025-02377-9

**Published:** 2025-04-27

**Authors:** James M. Mulqueeney, Thomas H. G. Ezard, Anjali Goswami

**Affiliations:** 1 School for Ocean & Earth Science, National Oceanography Centre Southampton, University of Southampton Waterfront Campus, Southampton, UK; 2 Department of Life Sciences, Natural History Museum, London, UK

**Keywords:** Morphometrics, Geometric morphometrics, Landmark-free morphometrics, Shape, Mammalia, Macroevolution

## Abstract

The study of phenotypic evolution has been transformed in recent decades by methods allowing precise quantification of anatomical shape, in particular 3D geometric morphometrics. While this effectiveness of geometric morphometrics has been demonstrated by thousands of studies, it generally requires manual or semi-automated landmarking, which is time-consuming, susceptible to operator bias, and limits comparisons across morphologically disparate taxa. Emerging automated methods, particularly landmark-free techniques, offer potential solutions, but these approaches have thus far been primarily applied to closely related forms. In this study, we explore the utility of automated, landmark-free approaches for macroevolutionary analyses. We compare an application of Large Deformation Diffeomorphic Metric Mapping (LDDMM) known as Deterministic Atlas Analysis (DAA) with a high-density geometric morphometric approach, using a dataset of 322 mammals spanning 180 families. Initially, challenges arose from using mixed modalities (computed tomography (CT) and surface scans), which we addressed by standardising the data by using Poisson surface reconstruction that creates watertight, closed surfaces for all specimens. After standardisation, we observed a significant improvement in the correspondence between patterns of shape variation measured using manual landmarking and DAA, although differences emerged, especially for Primates and Cetacea. We further evaluated the downstream effects of these differences on macroevolutionary analyses, finding that both methods produced comparable but varying estimates of phylogenetic signal, morphological disparity and evolutionary rates. Our findings highlight the potential of landmark-free approaches like DAA for large scale studies across disparate taxa, owing to their enhanced efficiency. However, they also reveal several challenges that should be addressed before these methods can be widely adopted. In this context, we outline these issues, propose solutions based on existing literature, and identify potential avenues for further research. We argue that by incorporating these improvements, the application of landmark-free analyses could be expanded, thereby enhancing the scope of morphometric studies and enabling the analysis of larger and more diverse datasets.

**Supplementary Information:**

The online version contains supplementary material available at 10.1186/s12862-025-02377-9.

## Background

Morphometrics, the quantitative analysis of shape, is a well-established family of methods in the field of biology [1]. In recent decades, geometric morphometrics has emerged as the gold standard for addressing evolutionary questions of shape in diverse datasets [2]. Typically, this approach relies on the manual placement of landmarks to produce two (2D) or three-dimensional (3D) coordinates by labelling homologous anatomical loci [3]. Raw coordinates are then transformed using methods such as Procrustes superimposition [4] to register objects to a common frame and isolate biological variation by minimising non-biological factors such as position, orientation, and size. Linear displacement across all the coordinates can then be measured and scaled by the number of landmarks to isolate the biological variation and estimate shape co(variation) [5].

Despite advancements in high-density morphometric techniques [6], including semi-automated placement of sliding semilandmarks [7], geometric morphometric methods remain largely manual. This makes them time-consuming and prone to observer bias which can lead to a lack of repeatability [8]. Moreover, with the increasing accessibility and affordability of high-resolution imaging [9, 10], alongside the development of tools for automated image segmentation [11, 12], databases of 3D images are expanding, providing vast amounts of data for morphometric analysis [13–15]. Thus, there is now a pressing need to improve the efficiency and resolution at which we capture shape variation to maximise the potential of this 3D data [16].

In addition to speed and repeatability, the requirement of homology for landmark placement, while important for biologically meaningful comparability across specimens, limits not only processing time but their applicability when comparing disparate taxa, as identifiable homologous points become more obscure and fewer in number, even within homologous structures [17]. Consequently, the reduction in the number of discernible landmarks when analysing phylogenetically distinct taxa results in the capture and comparison of only a minimal amount of variation, potentially leading to weaker biological inferences [18].

Emerging automated approaches in geometric morphometrics offer potential solutions to these challenges. Recent methods, including automated landmarking using atlas templates [19–21] or point clouds [22, 23] have allowed for improvements in efficiency. However, these techniques are still heavily tied to homology and may thus be less effective when attempting to overcome the current issues related to broad phylogenetic datasets. “Landmark or homology-free” approaches, which aim to capture shape data without relying solely on homologous landmarks, offer an alternative. These methods include psuedolandmarks [13, 24], iterative closest point (ICP) [25, 26], dense correspondence analysis [27–29], surface descriptors [30, 31] and large deformation diffeomorphic metric mapping (LDDMM) [32–34]. While some studies have demonstrated the promise of these methods at the interspecific level [35], most of these methods have primarily been tested on intraspecific datasets, leaving their utility at higher taxonomic levels undetermined.

Here, we focus on a LDDMM based method called Deterministic Atlas Analysis (DAA), implemented in the software *Deformetrica* [33, 34]. The DAA framework enables the comparison of shapes from images and meshes by using diffeomorphic transformations in 2D or 3D ambient space [32]. This comparison is achieved by quantifying the deformation required for a dynamically computed geodesic mean shape, known as an atlas [33], to fit each specimen in the dataset. Unlike some other diffeomorphic methods, DAA does not rely on a fixed template; instead, it iteratively estimates the optimal atlas shape by minimising the total deformation energy needed to map it onto all specimens [36, 37], meaning that results are sample dependent.

The DAA begins with the atlas generation process through selecting an initial template mesh, which undergoes geodesic registration [37] to represent the dataset under study. Following this, 2D or 3D deformations are computed to map the atlas onto each specimen in the dataset. The spatial extent of these deformations relative to the atlas is controlled by a kernel width parameter, with smaller values yielding finer-scale deformations. Based on this kernel width, a series of reference points, called control points, are generated. These points are initially evenly distributed within the ambient space surrounding the atlas, but as they increase, they are adjusted to fit areas with greater variability in the atlas. These act as the guides for shape comparison, eliminating the need for standard landmarks [33]. For each control point, a momentum vector (“momenta”) is calculated for each specimen in the dataset, representing the optimal deformation trajectory for aligning the atlas with each specimen. These momenta work within a Hamiltonian framework, derived from the velocity field of ambient space [34], and provide the basis for directly comparing shape variation. Techniques such as kernel principal component analysis (kPCA) [38] then facilitate the visualisation and exploration of covariation in the momenta-based shape data [39] (see Materials and Methods for further details).

In this study, we compare estimates of shape variation produced by DAA, following an adaption of the pipeline developed by Toussaint et al. [39], with those generated using manual landmarking and semi-landmarking techniques (hereafter referred to as manual landmarking for brevity). We compare the ability of each method to capture cranial shape across a broad and extensive dataset consisting of 322 crown and stem placental mammals [40], examining the influence of mesh modalities, atlas selection and the kernel width parameter on the results and the correlation between the two methods. Our comparison employs, Euclidean distances [41], the Mantel test [42, 43], and the PROcrustean randomisation TEST (PROTEST) [44, 45] to quantify the overall correlation between shape matrices. Heatmaps based on thin-plate spline deformations and Euclidean distance measures [46] are then used to identify how shape is captured differently using each method. Additionally, we investigate how the selection of method and kernel width influence downstream macroevolutionary analyses, including the estimation of phylogenetic signal, morphological disparity and evolutionary rates [47]. Together these metrics provide a comprehensive evaluation of how landmark-free approaches like DAA compare with traditional manual landmarking methods.

**Fig. 1:**
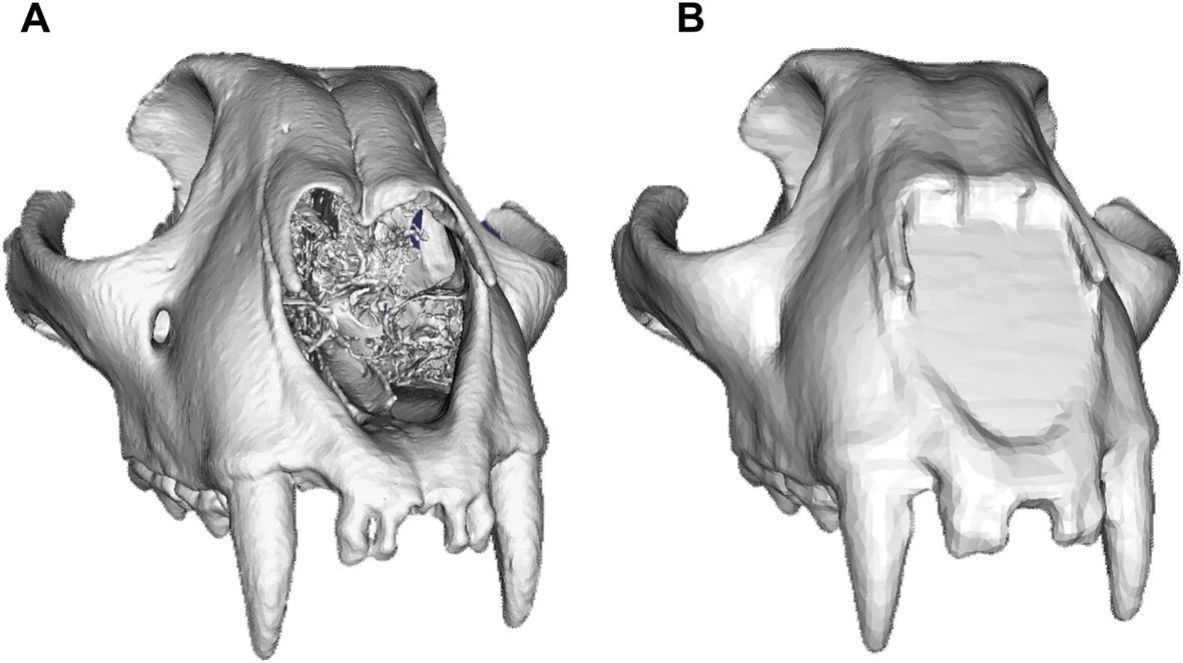
The process of transforming the (**a**) original mesh used in the “Aligned-only” analysis generated from computed tomography (CT) data into (**b**) a Poisson mesh requires the filling of holes using voxelisation and the use of a Poisson surface reconstruction algorithm [50] to generate watertight surfaces from orientated point sets. This allows to fill in the holes and evenly redistribute the faces and the vertices of the mesh prior to decimation. The effect here is demonstrated for the example of *Acinonyx jubatus*

Furthermore, as mesh topology has been suggested to influence the performance of landmark-free analyses [48, 49], we assess the impact of using both open and closed meshes (mixed modalities) generated from computed tomography (CT) and surface scanning, respectively in the same DAA (referred to as “Aligned-only”). Upon detecting an effect, we introduce the use of the “Poisson” mesh (Fig. 1) generated using Poisson surface reconstruction [50], which creates watertight, closed meshes as a solution to overcome the issues of mixed modality datasets. Our comparative analyses capture the effects of all these aspects to provide a clear understanding of the considerations and potential utility of this approach for diverse applications.

## Results

### Comparison of initial template selection on atlas generation

Before extensively performing DAA, an initial template for the atlas generation process must be selected. As this choice could influence the results [51–53], we tested multiple initial templates based on the results of the manual landmarking analysis (see Materials and Methods). Our analysis indicated that initial template selection had a minimal overall impact on shape predictions (Additional File, Figures A1 and A2, Data A1-3). Using a fixed kernel width of 20.0 mm, we found that the results obtained from different templates were highly correlated, with the *Arctictis binturong* template showing a strong and significant correlation with both the *Cacajao calvus* (*R*^2^ = 0.957, *p* < 0.05) and *Schizodelphis morckhoviensis* (*R*^2^ = 0.801, *p* < 0.05) atlases (Figure A2).

Any differences were primarily attributed to the varying number of control points generated by each template: *A. binturong* yielded 270 control points, *C. calvus* 420 control points, and *S. morckhoviensis* only 32 control points. However, we observed a systematic bias for *C. calvus* and *S. morckhoviensis*, where the template specimen, which would typically cluster with those at the morphological extremes, was instead drawn toward the centre of the kernel principal component analysis (kPCA) plots. This artefact reduced morphological differentiation by shifting the template specimen away from its morphologically similar counterparts. Based on these observations, *A. binturong* was chosen as the initial template for all subsequent analyses.

### Comparison of aligned-only and Poisson meshes

**Fig. 2:**
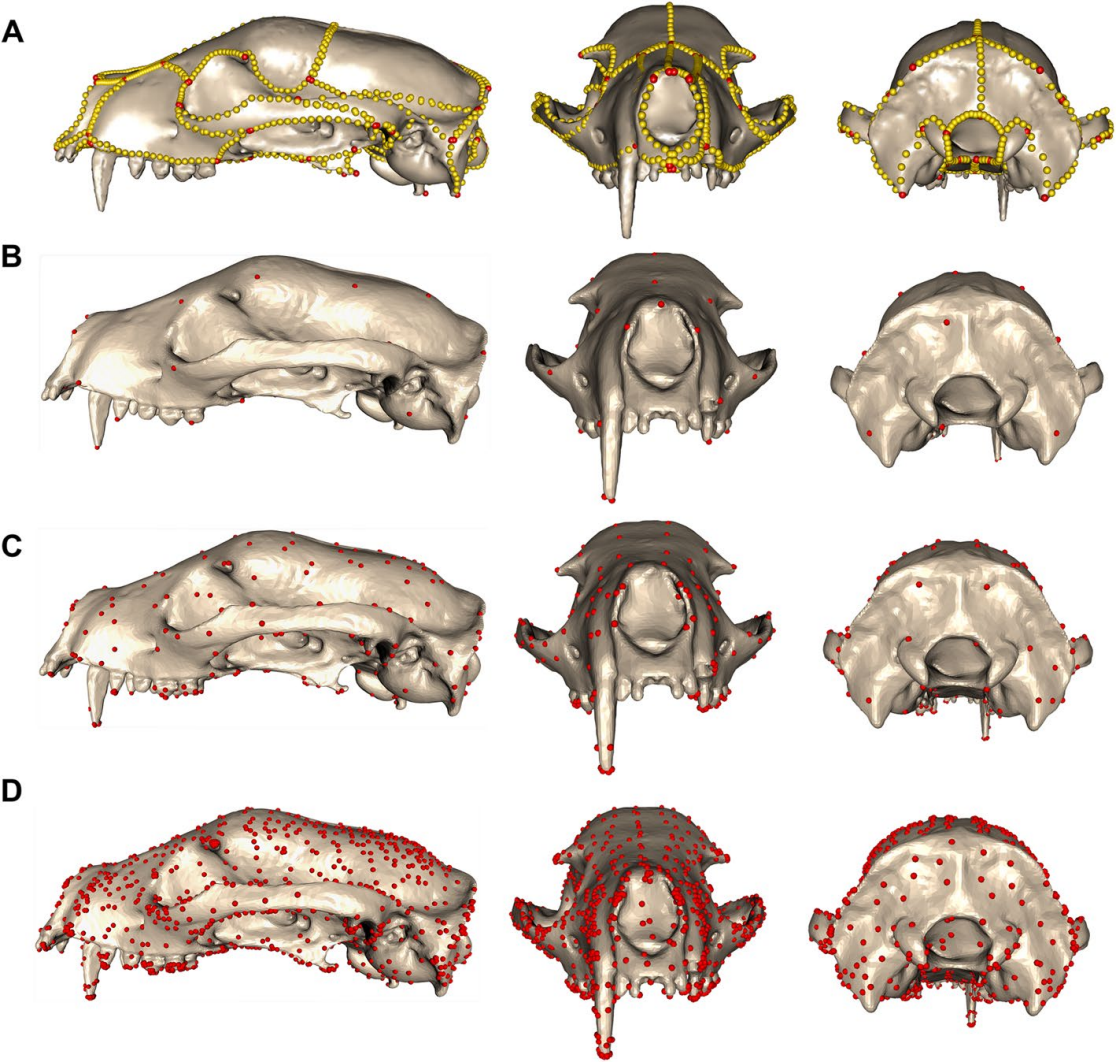
Comparative assessment of morphometric methods applied to the 3D mesh of the atlas specimen *Arctictis binturong* (MNHN 1936-1529). The figure contrasts (**a**) a manual landmarking approach using 754 landmarks and sliding semilandmarks with mapped control points through Deterministic Atlas Analysis (DAA) under different kernel widths: **b** 40.0 mm, generating 45 control points; **c** 20.0 mm, producing 270 control points; and (**d**) 10.0 mm, resulting in 1,782 control points

Adjusting the kernel width parameter in the DAA alters the spatial extent of the neighbouring points based on a Gaussian kernel that corresponds to the selected initial template (see Materials and Methods). When using *A. binturong* as the initial template, the kernel widths of 40.0 mm, 20.0 mm, and 10.0 mm produced 45, 270 and 1,782 control points, respectively (Fig. 2, Additional File, Figure A3). For each of these control points, a momentum vector (“momenta”) which describe the optimal deformation trajectory of each specimen to fit the atlas were calculated for all 322 specimens and used to compare the overall shape variation with those obtained using manual landmarking.

Initial comparisons showed discrepancies when comparing the Aligned-only dataset (comprising open and closed surfaces) and the Poisson mesh dataset (all watertight closed surfaces) in the DAA. For the Aligned-only dataset, results across all three kernel widths showed an artificial distinction between specimens with open surfaces (from CT scans) and those with closed surfaces (from surface scans) across each of the evaluated principal component (PC) axes (Additional File, Figure A4 and A5 and Data A4-6). This is exemplified in PC1, where specimens with open surfaces clustered towards the positive end, while those with closed surfaces grouped at the negative end.

**Fig. 3:**
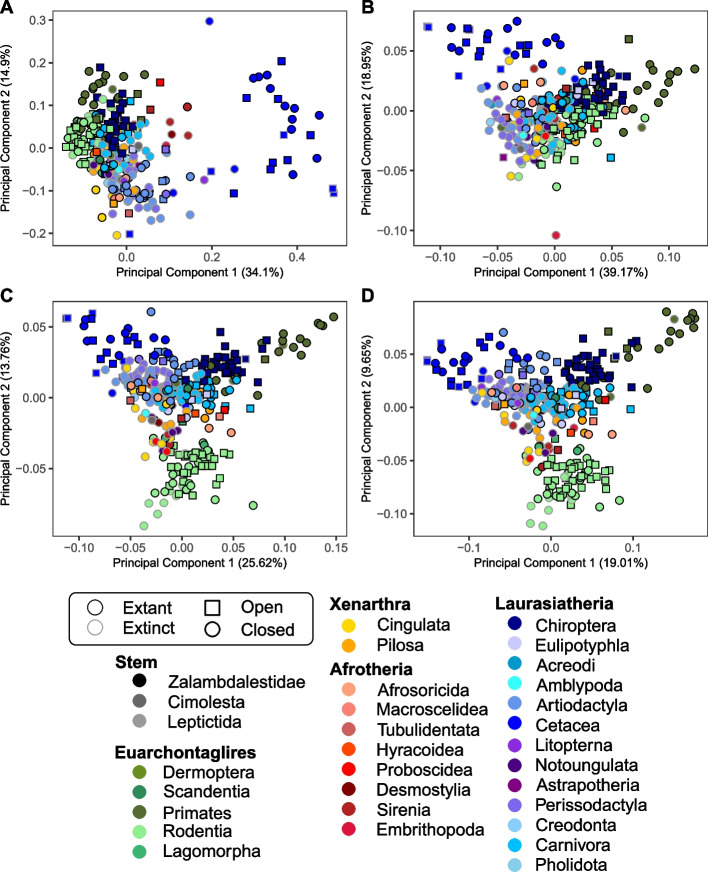
Principal component plots (PC) for PC1 and PC2 obtained for the shape analysis using the “Poisson” meshes, comparing the results between (**a**) manual landmarking with 754 landmarks and sliding semilandmarks and the DAA method using kernel widths of (**b**) 40.0 mm, yielding 45 control points, (**c**) 20.0 mm, yielding 270 control points, and (**d**) 10.0 mm, yielding 1,782 control points

Standardising the data using the Poisson mesh redistribution [50] to generate watertight closed meshes for all specimens helped to mitigate the impact of mixed modalities on the shape estimates. With the Poisson meshes, the artificial separation along PC1 was eliminated, and specimens clustered more closely with others of the same taxonomic order, rather than the mesh type (Fig. 3, Additional File, Figure A6 and Data A7 - 9). The removal of the impact of mixed modalities also led to a decrease in the percentage of variation explained by the first PC axis.

**Fig. 4:**
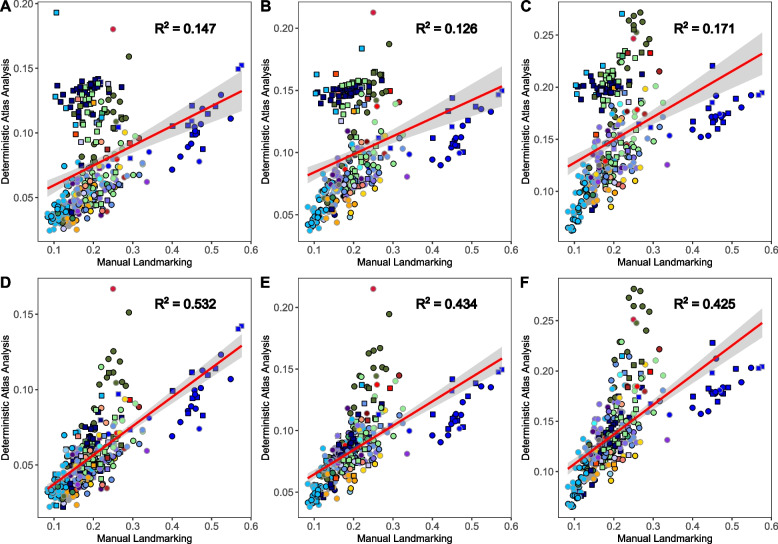
Pairwise Euclidean distance comparisons between each specimen and the atlas, *Arctictis binturong* (MNHN 1936-1529) measured across 321 axes. Distances are shown for the manual landmark data and “Aligned-only” meshes using Deterministic Atlas Analysis (DAA) with a kernel width of (**a**) 40.0 mm producing 45 control points, (**b**) a kernel width of 20.0 mm producing 270 control points, and (**c**) a kernel width of 10.0 mm producing 1,782 control points. Similarly, distances for Poisson meshes are displayed for (**d**) a kernel width of 40.0 mm producing 45 control points, (**e**) a kernel width of 20.0 mm producing 270 control points, and (**f**) a kernel width of 10.0 mm producing 1,782 control points. The red line represents the correlation between values with a 95% confidence interval. All correlations were significant (*p* < 0.01)

The changes in PC1 when removing the impact of mesh modality were also clear when comparing the correlation of Euclidean distances [41] for each DAA with the manual landmarking results (Fig. 4). Although all six DAA analyses (three Aligned-only, three Poisson meshes) showed a significant correlation with the manual landmarking result (*p* < 0.05, Fig. 4, Additional File, Table A1), there was marked improvement in the correlation from the Aligned-only dataset (M = 0.148, SD = 0.023) to the Poisson mesh dataset (M = 0.460, SD = 0.059; t(2.02) = 8.305, *p* < 0.05). In the Aligned-only dataset, specimens with open surfaces were separated from those with closed surfaces, unlike in the Poisson mesh analysis. This realignment of specimens when using Poisson meshes contributed to the improved correlation between manual landmarking and DAA (Fig. 4).

When comparing the correlation between Euclidean distances measured for each specimen from *A. binturong* in the Aligned-only and Poisson mesh analyses across major orders (> 10 specimens in analysis; Additional Material, Data A10), we observed significant improvements for all orders except Perissodactyla*,* Pilosa*,* and Primates*,* where no statistically significant decrease was found (*p* > 0.05). The effect of Poisson redistribution was particularly pronounced in groups with a higher proportion of CT scans, notably Chiroptera*,* Rodentia*,* and Carnivora. In these groups, the mean R^2^ (averaged across kernel widths) increased significantly (*p* < 0.05) by 0.60, 0.50, and 0.38, respectively.

Further comparisons of the shape matrices obtained from the manual landmarking and DAA, performed using the Mantel test [42, 43] and the PROcrustean randomisation TEST (PROTEST) [44, 45], also demonstrated the enhanced correspondence when using Poisson meshes (Table 1). The Mantel test showed a significant improvement in the Mantel r statistic (where 0 shows no correlation and 1 shows perfect correlation) from the Aligned-only dataset (M = 0.285, SD = 0.0229) to the Poisson mesh dataset (M = 0.619, SD = 0.0354; t(2.02) = 11.384, *p* < 0.05). The Procrustes sum of squares (where 0 indicates no difference and 1 indicates a significant degree of dissimilarity) were significantly decreased from the Aligned-only dataset (M = 0.679, SD = 0.0778) to the Poisson mesh dataset (M = 0.450, SD = 0.0461; t(2.02) = − 9.0064, *p* < 0.05) and the Procrustes root mean squared error (larger value shows more error) were also decreased from the Aligned-only dataset (M = 0.0459, SD = 0.00262) to the Poisson mesh dataset (M = 0.0374, SD = 0.00189; t(2.02) = − 10.652, *p* < 0.05). Due to these results, all further comparisons were exclusively conducted using the Poisson meshes.

**Table 1:** Correlation comparisons between manual landmarking result and DAA for both the “Aligned-only” and “Poisson” meshes. The results show the correlations between matrices evaluated using the Mantel Test [42, 43], with results demonstrated using the Mantel r statistic, where a value of 0 shows no correlation, and 1 shows a perfect correlation, the Procrustes sum of squares, where a value of 0 indicates a perfect fit and 1 indicates high dissimilarity, and the Procrustes root mean squares estimates (high value means more error) using the PROcrustean randomisation TEST (PROTEST) [44, 45]. The correlation between the percentages of variation (eigenvalues) across each of the 321 axes is also given where an R^2^ value of 1 indicates a perfect fit. All results are significant (*p* < 0.01), with the best values for each statistic being highlighted in bold

**Data Set**	Mantel r statistic	**Procrustes sum of squares**	**Procrustes root mean squared error**	**Eigenvalue correlation (R**^**2**^**)**
Aligned-only 40	0.2799	0.762	0.049	0.942
Aligned-only 20	0.2647	0.666	0.045	0.962
Aligned-only 10	0.3098	0.608	0.043	0.975
Poisson 40	**0.6574**	0.503	0.040	**0.993**
Poisson 20	0.6107	**0.418**	**0.036**	0.978
Poisson 10	0.5879	0.430	0.037	0.962

### Comparison between manual landmarking and landmark-free methods

Examining the patterns of shape variation across the first four PC axes (Fig. 3, Additional File, Figure A6) revealed both differences and similarities between the manual landmarking and DAA results. The most notable differences were observed for Cetacea and Primates. In the manual landmarking analysis (Additional File, Data A11), cetaceans in PC1 were found far away from the other clades but were positioned much closer in the DAA, while still occupying the extreme values along PC1. Conversely, primates plotted much closer to the other taxa in the plot of the first two PC axes in the manual landmarking approach but were more distinct in the results of the DAA, especially when using a higher number of control points. Rodents consistently formed a distinct cluster across all analyses, but this separation was more pronounced in the DAA. Notably, in the 20.0 mm and 10.0 mm kernel width analyses, the rodent cluster shifted to PC2 rather than PC3, whereas it remained on PC3 for the 40.0 mm analysis. A key similarity across methods and kernel widths was the consistent clustering of carnivorans and artiodactyls in the central regions of the PC plots.

When comparing the results across all PC axes for the three DAA using Poisson meshes (Fig. 3, Additional File, Figure A6 and Data A7-9), we found a significant but moderate correlation with the manual landmarking results across all three kernel widths when measuring Euclidean distances amongst specimens (Fig. 4, Additional File, Table A1). Notably, we observed a decline in the correlation between the manual landmarking and DAA as the kernel width decreased (the number of control points increased), with the most pronounced decline occurring when going from 40.00 m to 20.0 mm (R^2^ decreased by 0.098). Removing both Cetacea and Primates from these comparisons yielded contrasting results. For the largest kernel width (40.0 mm), excluding these orders reduced the R^2^ value from 0.532 to 0.384. In contrast, for the smaller kernel widths of 20.0 mm and 10.0 mm, the R^2^ values increased from 0.434 to 0.523 and from 0.425 to 0.525, respectively.

Reviewing the correlation in Euclidean distances within each of the major orders (> 10 specimens in the dataset; Additional Material, File A7), showed contrasting patterns across different numbers of control points. For instance, the highest correlation with manual landmarking was observed at a kernel width of 40.0 mm for Artiodactyla*,* Cetacea*,* and Pilosa. In contrast, all other orders: Carnivora, Chiroptera, Perissodactyla, Primates and Rodentia showed improved correlation as the kernel width decreased. The magnitude of these changes varied substantially between groups, with Carnivora showing an increase in correlation of 0.355 and Pilosa a decrease of 0.50, while the correlation for Cetacea only varied by 0.08.

Further assessments of the impact of kernel width on the correlation between DAA and manual landmarking, performed using the Mantel test, also showed a significant but moderate correlation across all three kernel widths (Table 1). These correlations were slightly higher than those obtained using Euclidean distances (Fig. 4, Additional File, Table A1), although the maximum value of correlation remained moderate at 0.657. Consistent with previous results, correlation values decreased as kernel width decreased and the number of control points increased (Table 1), with the most pronounced decline occurring between 40.0 mm and 20.0 mm. The results from PROTEST again showed significant correlations, but here we found that a kernel width of 40.0 mm had the worse fit to the data, and 20.0 mm the best. The variation explained by each of the axes were also highly correlated and conserved across each analysis (Table 1, Additional File, Data A12-17).

### Comparisons of estimated mean shape using heatmaps

**Fig. 5:**
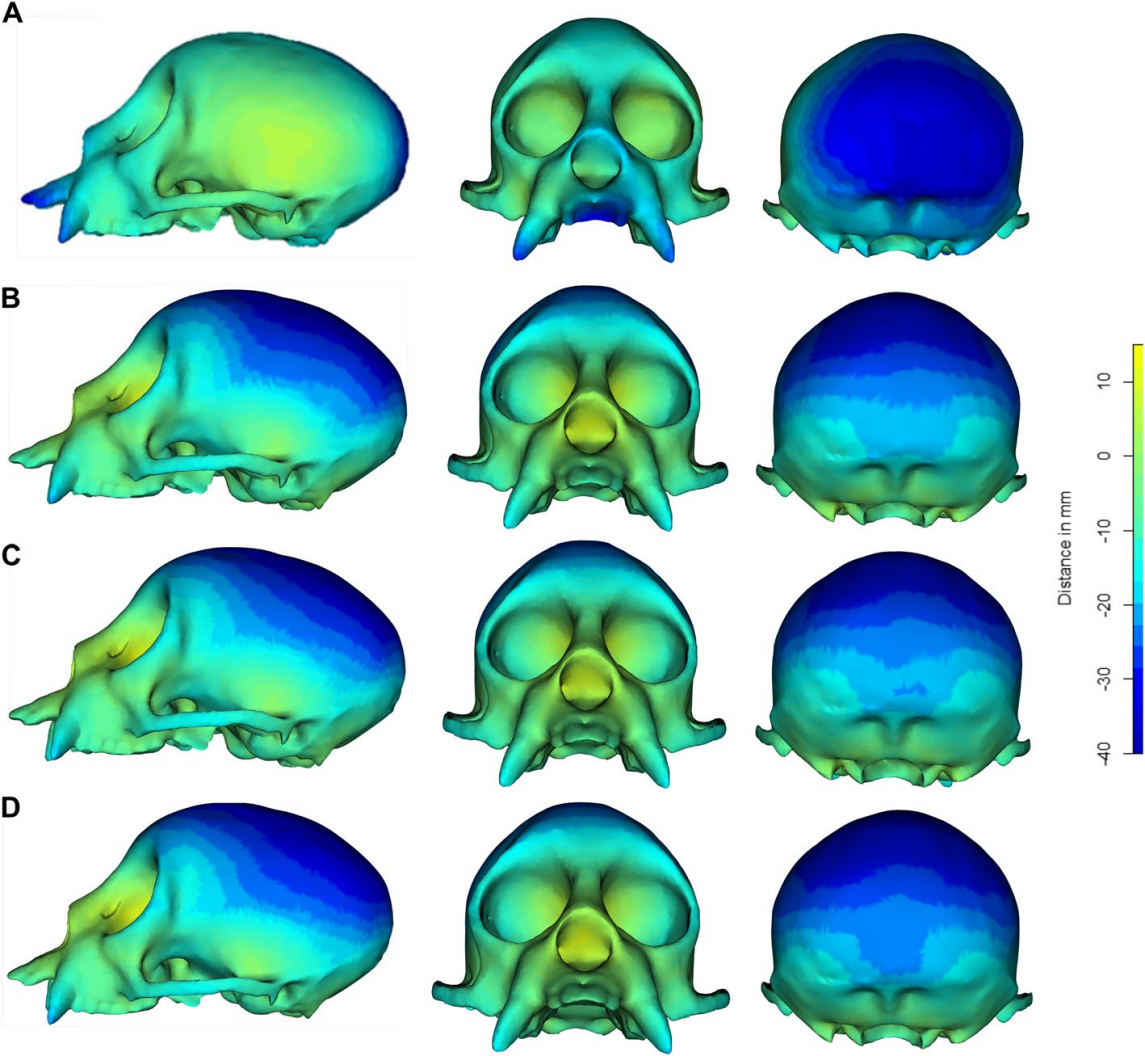
Displacement heatmaps for the specimen, *Cacajao calvus* (NHMUK ZD 1928.4.27.6) generated by comparing its shape with the estimated mean shape of the 322 specimens across each analysis using Euclidean distances measured using the *meshDist* function in Morpho [46] v.2.12. Comparisons are shown for distances of the vertices in the *C. calvus* mesh and mean shape estimated in (**a**) the manual landmarking scheme with 754 landmarks and sliding semilandmarks, and for the geodesic mean shape estimated in the Deterministic Atlas Analysis (DAA) with (**b**) a kernel width of 40.0 mm producing 45 control points, (**c**) a kernel width of 20.0 mm producing 270 control points, and (**d**) a kernel width of 10.0 mm producing 1,782 control points. The heatmaps demonstrate the differences in where shape variation is captured for both methods

Comparative visualisations of how shape variation was captured using manual landmarking and DAA were generated for a single specimen of the eight major orders (Fig. 5, Additional File, Figure A7). To do this, we produced heatmaps based on Euclidean distances measured between corresponding vertices (see Materials and Methods). Viewing the heatmaps revealed some similarities, but overall marked differences in how each method captured variation. For instance, when looking at *C. calvus* (Fig. 5)*,* both methods identified substantial negative displacement in the occipital region and positive displacement in the eye sockets relative to the mean shape. On the other hand, the manual landmarking approach detected minimal variation in the parietal region**,** whereas the DAA indicated significant negative displacement in this area. This pattern was consistent among other specimens at the positive end of PC1 in the DAA, whereas those at the negative end of PC1 (e.g. Cetacea, Artiodactyla) showed reduced overall displacement in the DAA compared to the manual landmarking approach, especially in the nasal and premaxilla regions.

### Comparison of effects on downstream macroevolutionary analyses

After assessing the differences in shape variation estimates, we evaluated their impact on downstream macroevolutionary analyses. All datasets showed significant phylogenetic signal when measuring using K_mult_ [54] (*p* < 0.01, Table 2), with the manual landmarking approach yielding the highest value, indicating that shape correlated with phylogenetic relationships across all analyses. In the DAA, we found that the phylogenetic signal consistently decreased as the kernel width decreased (as the number of control points increased, Table 2).

**Table 2:** Comparison of phylogenetic signals measured using the *physignal* function applying the K_mult_ statistic [54] through 99 iterations using geomorph [47] v.4.05 in R. All values are statistically significant (*p* < 0.01)

**Dataset**	**Phylogenetic Signal**
Manual	0.494
Poisson 40	0.477
Poisson 20	0.405
Poisson 10	0.303

**Fig. 6:**
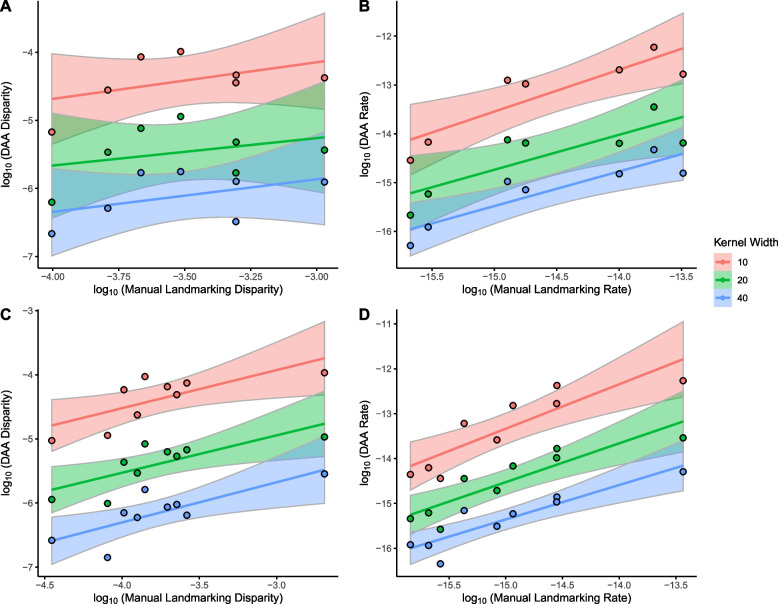
Comparisons of estimates on a logarithmic scale for (**a**, **c**) morphological disparity and (**b**, **d**) net rates of shape evolution based on (**a**, **b**) dietary categories and (**c**, **d**) locomotion modes, between the manual landmarking data containing 754 landmarks and sliding semilandmarks and Deterministic Atlas Analysis (DAA) with kernel widths of 40.0 mm producing 45 control points, 20.0 mm producing 270 control points, and 10.0 mm producing 1,782 control points, calculated using the *morphol.disparity* and *compare.evol.rates* functions in geomorph [47] v.4.05. Each line represents the line of best fit with 95% confidence intervals

Next, we estimated morphological disparity and evolutionary rates for specific ecological categories and compared the results between the manual landmarking and each DAA dataset (Fig. 6, Additional File, Table A2, and Data A18-19). We focused on the ecological categories of diet and locomotion as these can be reliably estimated for fossils and thus had values available for all specimens. For diet, we found no significant correlation across any of the kernel widths for the measures of morphological disparity (*p* > 0.05) but did find highly significant values for correlations for evolutionary rate (*p* < 0.05), particularly for the kernel width of 10.0 mm when comparing DAA to manual landmarking. For locomotion, we recovered significant correlations for both measures of morphological disparity and evolutionary rates (*p* < 0.05), with the highest values of correlation found for the kernel width of 20.0 mm when compared with manual landmarking.

## Discussion

To advance morphometrics into the era of big data, it is essential to develop methodologies that efficiently capture precise, high-dimensional morphological data across extensive and diverse datasets. However, this remains difficult due to the characteristics of current geometric morphometric approaches, especially their time intensive nature and lack of applicability to disparate datasets. In this study we sought to investigate if landmark-free methods, such as the Deterministic Atlas Analysis (DAA), can overcome these bottlenecks. Our analysis demonstrates that these methods are significantly quicker to apply, with the processing of all 322 meshes and analysis taking ~ 120 hours compared to the ~ 750 hours required for expert manual labelling. When considering only the analysis using DAA excluding time spent on the generation of Poisson meshes (which may not be necessary if a single imaging approach is used, i.e. only CT scans), the time for generation of morphometric data is reduced from ~ 750 hours down to eight hours. This improvement in efficiency is very promising, allowing for large-scale morphological studies such as the one performed here to be conducted in the fraction of the time, thus significantly improving the scalability of geometric morphometric approaches.

The results of manual landmarking and DAA were almost all significantly correlated, including the comparisons between shape and the downstream macroevolutionary analyses, but often these relationships were only moderate. The measured Euclidean distances showed correlations of 55% or lower, while the Mantel test reached a maximum of 65% (Table 1). This suggests that at least 35% of shape variation captured by one method remains unexplained by the other. Additionally, the sensitivity of results to changes in the kernel width parameter indicates that spatial scale plays a crucial role in shaping the results. Here, we attempt to disentangle the major reasons for the similarities and differences between the results, and present suggestions to help maximise the potential of landmark-free methods in capturing accurate shape variation in the future.

### Comparison of template selection on atlas generation

As previous studies have shown that the initial template selection can impact results [51–53], we began our study by testing three morphologically disparate taxa as our input template. In contrast to these previous findings, we found that the results remained highly consistent across templates, with the primary source of variation in the results arising from differences in the number of control points generated for each selected specimen, despite using a fixed kernel width (Additional File, Figure A1 and A2, Data A1-3). While these templates represented a single specimen, the DAA approach was shown to mitigate this impact, which we attribute to the reduction in total deformation energy due to the construction of a new mean shape based on geodesic transformations [36, 37]. Since the dataset remained constant across analyses, these transformations ensured consistency across different templates.

Although impact was minimal, we still suggest that the specimen closest to the estimated mean shape in the manual landmarking analysis to be the most optimal, in this case, *A. binuturong*. This is because when using specimens with more unusual morphologies among the dataset (*C. calvus and S. morckhoviensis*) we found that there is a tendency to pull the template towards the centre of the principal component (PC) plots (Additional File, Figure A1). This phenomenon has also been observed in other automated shape analysis techniques including automated landmarking methods [21, 55]. Since this pipeline requires Type 1 manual landmarks to pre-process the data, we recommend future users visualise the shape predictions of these landmarks to help guide the template selection.

### Comparison of aligned-only and Poisson meshes

Previous research on landmark-free methods has emphasised the importance of using meshes and surfaces with consistent geometry and topology to achieve accurate results [48, 49]. Our findings support this conclusion, as the DAA approach was significantly influenced by the mesh modalities used. Unlike manual landmarking approaches, which can more readily accommodate mixed mesh types [35, 56], landmark-free methods such as DAA may require standardisation when incorporating meshes with both open and closed surfaces. In our study, combining CT scans (open surfaces) and surface scans (closed surfaces) without additional processing led to inaccuracies in the measured shape variation due to biases associated with mesh modality (Additional File, Figure A4 and A5, and Data A4-6).

To address this issue, we applied voxelisation and Poisson surface reconstruction [50] to generate Poisson meshes with watertight, closed surfaces (Fig. 1). This standardisation effectively eliminated the impact of mesh modality on the overall results (Fig. 3, Additional File, Figure A6, and Data A7-9), facilitating the integration of different data types within the same analysis. By removing the artefactual differences between mixed modalities, this approach significantly improved the correlation between DAA and manual landmarking methods. We, therefore, recommend, when possible, these techniques should be applied, or analyses be performed only using a single mesh type (e.g. only surface scan or only CT-scans).

### Comparison of landmark-free and manual landmarking approaches

The significant but moderate correlation and low effect sizes (Fig. 4) between results obtained through manual landmarking and DAA, suggest that landmark-free methods can capture some aspects of shape variation captured in the manual landmarking approach, but also indicates that marked differences exist. The most notable similarity of the approaches was the ability to capture the classic dolichocephalic-to-brachycephalic trend (variation in skull elongation vs. breadth) [57], though the pattern was observed along PC1 in the DAA approach and PC2 in the manual landmarking analysis (Fig. 3, Additional File, Figure A6, and Data A7-9). This indicates that both methods are sensitive to varying lengths and widths of the crania but also implies that both methods prioritise different aspects of shape variation.

The differences in how shape is captured between the two approaches can be best highlighted by examining the changes in the measured Euclidean distances [41] (Fig. 4) and heatmaps (Fig. 5). From the former, we found that the cetaceans had reduced distances from the atlas in the DAA compared to manual landmarking, whilst primates exhibited greater distances. Examination of the heatmaps (Additional File, Figure A7) and aligned specimens (Additional File, Figure A8) suggest that these differences can be attributed to two main factors, the first is differences in scaling and the second is the way that measurement points are placed along the crania (Fig. 2, Additional File, Figure A3).

The difference in scaling is evident from the reduced negative displacement in the heatmaps for cetaceans (Additional File, Figure A7) and is further showcased when comparing the scaling of *S. morckhoviensis* in manual landmark and landmark-free approaches against the mean shape estimated in both sets of analyses (Additional File, Figure A8). These scaling discrepancies primarily affect the premaxilla and nasal regions, which appear less distinct in DAA compared to manual landmarking because of the cetaceans being scaled to a smaller size (Additional File, Figure A7 and A8). The likely causes of these differences are twofold. First, because scaling in DAA is applied to the mesh based on the original landmarks, morphological regions not captured by landmarks are not considered in the scaling process. Second, while manual landmarking allows for relative bone sizes within the cranium to be explicitly considered, the DAA mesh-based scaling does not capture these relationships among elements.

The lack of representation of relative bone sizes and positions within the crania are also likely important factors that lead to differences in how shape variation is captured. For instance, the DAA approach appears to struggle in capturing telescoping in the cetaceans (where cranial bones overlap), leading to reduced differentiation. As such, the absence of the midline convergence in the parietal bones within the cranial vault is not detected and the changing of these bones relative to another cannot be captured using this approach. By contrast, DAA increases the differentiation of primates from the other taxa by capturing greater variation in the cranial vault and brain case, which are sparsely sampled in the manual landmarking approach. This is highlighted in the heatmaps of *C. calvus* (Fig. 5), where there is increased displacement captured in the cranial vault and parietal bones. Similarly, rodents appear more distinct in the first two PC axes in the DAA, which we attribute to an increased emphasis on the incisor region, which is less covered in terms of spatial points in the manual landmarking approach. The inability to capture the cranial vaults and incisor regions is not an inherent limitation in the manual landmarking approach, but rather in this implementation. The landmark scheme used here which uses landmarks and curve sliding semilandmarks placed on sutures is applied to a phylogenetically broad dataset, leaving anatomical regions such as the cranial vaults and incisors without measurement points. To alleviate such issues, studies have sought to use a combination of fixed landmarks and surface sliding semilandmark patches [7, 58], but these methods are often difficult to reproduce, time-consuming and thus are rarely applied to large datasets. Morever, these techniques become more difficult to apply when comparing more disparate taxa, as overlapping homologous regions become harder to differentiate [6].

On the other hand, the DAA approach more readily provides this extensive spatial coverage with the number of control points easily being adjusted using the kernel width parameter. A key advantage of DAA is that this spatial coverage is both uniform and automated rather than user-selected, reducing biases associated with the pre-selection of specific morphological regions [59]. This standardisation ensures more consistent capture of shape variation, reducing the risk of missing or inadequately covering critical anatomical areas, a limitation observed in the manual landmarking analysis conducted here. Consequently, the expanded spatial extent facilitated by DAA may result in more accurate and comprehensive comparisons of morphological shapes.

This uniformity also presents challenges, particularly when anatomical variation is extensive and the overlap of homologous regions becomes less distinct. Since control points are not constrained by specific anatomical landmarks, there is a risk of unintended blurring of anatomical regions, potentially leading to inaccuracies. This issue is especially pronounced when comparing specimens with large appendages, such as horns or teeth, to those without, or when analysing specimens with missing anatomical features, such as the postorbital bar or zygomatic arches, alongside those that retain them.

These effects are evident in the results: as the number of control points increases, correlation in the Euclidean distances measured for within Artiodactyla (a group with prominent horns) declines from 0.436 to 0.358. Similarly, in Pilosa, where two-thirds of specimens lack zygomatic arches, correlation drops sharply from 0.588 to 0.0351 (Additional File, Figure A7). Unlike manual landmarking, DAA does not allow users to adjust measurements to account for such anatomical anomalies, likely contributing to the lower correlation between the two methods. Our results show that this effect becomes more pronounced as the number of control points are increased. However, it is worth noting that landmark-based approaches generally exclude structures that vary in presence entirely, meaning that regions that contribute to significant variation are left out of analyses, although methods that account for variably present regions with semilandmarks is discussed in Bardua et al. [7].

### Effects on macroevolutionary analyses

All measured values of phylogenetic signal across both methods were statistically significant, but we observed a decline when transitioning from the manual landmarking approach to the DAA, with the most prominent decline occurring in the analysis using the largest number of control points (smallest kernel width, Table 2). This decline in this dataset likely results from capturing variation beyond biologically homologous structures [60], something that becomes more prominent as spatial resolution increases. While a greater number of control points enhances spatial coverage, it also introduces noise due to the proximity of measured points [5] and the increased placement of points in regions such as teeth, horns, and nasal cavities which are likely less consistently present or preserved across specimens (Fig. 2). Furthermore, missing (as opposed to absent) structures such as the delicate zygomatic arch or pterygoid in mammal skulls, presents a greater challenge in DAA, especially at higher spatial resolutions, as it artificially amplifies non-biological differences between individuals with complete crania and those with missing structures.

Comparisons between the values of morphological disparity (Fig. 6, Additional File, Table A2) obtained through manual landmarking and DAA consistently show lower magnitudes for the latter. However, as the kernel width decreases (and control points increase), the magnitude of disparity in DAA increases, with values for the 10.0 mm kernel width becoming more comparable to those from manual landmarking. The higher values in manual landmarking are likely due to the selective placement of landmarks, which amplifies shape differences among individuals. In contrast, DAA, with its increased spatial resolution, gradually recovers these differences as more control points are introduced. On the other hand, the magnitude of the values obtained for the phylogenetic rates (Fig. 6, Additional File, Table A2), was less affected by the selection of method and kernel width. This discrepancy likely stems from the fact that the measures of disparity are absolute values, while evolutionary rates are relative measures, comparing values within one class to another [47].

The levels of correlation between the measures of morphological disparity and evolutionary rates also show some contrasts. The correlation for the evolutionary rates for both diet and locomotion were both high and significant (Table A2), but only the latter was significantly correlated for morphological disparity. The lower levels of correlation in the morphological disparity measured for the diet are likely due to the presence of multiple classes that are occupied solely by cetaceans, which exhibit markedly different results between the manual landmarking and DAA and are on the extreme values of the distribution. This difference leads to greater differences in these classes, resulting in a reduction in the overall correlation.

The retention of significant phylogenetic signal and the similarity in evolutionary rate estimates between manual landmarking and DAA with a larger kernel width suggest that DAA captures biologically meaningful patterns. While using an excessive number of control points may sometimes weaken biological inferences, particularly when analysing incomplete specimens or those with significant morphological differences, it also provides enhanced spatial resolution. This higher resolution allows for the detection of subtle morphological variations that might otherwise be overlooked in manual landmarking, facilitating deeper insights into both phylogenetic and ecological dynamics.

### Future improvements and applications for landmark-free approaches

Our results highlight that landmark-free approaches are promising but further improvements are important for achieving the consistency and biological interpretability of analyses using manual landmarking methods. Many of the shortcomings of DAA were initially observed in manual landmarking methods, but have since been addressed. For example, within the dataset here, the inclusion of fossil data led to having incomplete specimens that had missing anatomical structures. As noted previously, for groups such as Pilosa which had few complete specimens, the correlation with the manual landmarking data was lower, and progressively decreased as a larger number of control points were used. Furthermore, specimens with missing anatomical features, such as *Euhapsis ellicottae*, produced markedly different patterns of shape variation (Fig. 3). Unlike DAA, manual landmarking approaches can mitigate these issues through strategies such as missing data estimation [61] or interpolating missing landmarks [62], and even without these interventions, manual approaches tend to produce less confounded results [35, 56, 63]. Solutions to overcome these issues in DAA are likely to come from emerging deep learning approaches, some of which have shown promise in predicting missing anatomical regions [64].

Another important consideration is the landmark-free methods like DAA often include anatomical features that would typically be excluded from manual landmarking analyses. In this dataset, features such as horns and teeth were captured, which impacts shape comparisons by inflating the differences between specimens. For instance, when using when using a larger kernel width (fewer control points), specimens with large horns, such as *Arsinoitherium zitelli*, and large teeth, such as *Odobenus rosmarus*, plotted in distinct areas of PC1, separate from the rest of their clade (Fig. 3). This effect is less obvious when using a higher number of control points, but the increased capturing of these areas also likely contributes to the decreased correlation between methods. The inclusion of such features can be seen as either an advantage or a drawback, depending on the question, dataset and variation of interest for an analysis.

One area where DAA could be enhanced for more robust shape comparisons that retain homology is by applying it to isolated individual skeletal elements, for example each bone within the cranial structure. Applying DAA to structures made of multiple elements, impedes studies on the independent evolution of specific elements [65] and their relationship through phenotypic integration and modularity [66, 67], and may also prevent obtaining accurate mesh scaling and comparisons between taxa. In contrast, manual landmarking techniques that capture regions along suture lines help ensure the retention of homology, which likely explains why groups like Cetacea may appear more disparate from other taxa in the manual landmarking analysis than DAA. We, therefore, suggest that parcellating meshes into different anatomical regions using computer vision techniques such as SPROUT [68], MeshCNN [69] or BounTI [70] and then applying DAA to the individual elements may help improve the biological interpreation of comparisons by focusing on homologus elements. Additionally, the ability to assign control points to defined anatomical regions, could help to more accurately capture biological variation and better preserve homology in the future use of landmark-free approaches. As noted above, the application of DAA to complex structures or individual elements will depend on the question or dataset of interest.

### Implications for future studies

Transitioning from manual landmarking to automated approaches, whether through automated landmarking or landmark-free methods such as DAA presents significant challenges. Nevertheless, only through rigorous testing, such as the analyses conducted here, can we refine these methods and extend their applications. If biologically meaningful results can be consistently obtained, as our analyses suggest is possible, these approaches hold the potential to be transformative in the field. They will help to reduce manual data handling, which will reduce both inter- and intra-operator errors commonly associated with manual landmarking studies [71, 72]. The efficiency of extracting shape variation will also be significantly improved, allowing large-scale analyses that would traditionally take months or years to be completed in weeks or days. In an era of rapdily expanding phenotypic datasets, driven by increasing acessibility of 3D imaging technologies [73], the ability to swiftly and accurately analyse morphological variation is crucial.

Despite these advantages, methodological considerations remain. One of the primary challenges in landmark-free approaches such as DAA is the shift away from fixed, unambiguously homologous points for shape comparisons. While manual landmarking has long been the gold standard, its reliance on a limited set of homologous points, especially across phylogenetically distant taxa, can be both a strength and a limitation [59]. On the one hand, it ensures biological validity by limiting analyses to directly comparable (i.e. homologous regions) anatomical regions. On the other hand, it restricts spatial resolution and may exclude aspects of morphological variation that are relevant to broader evolutionary questions.

The potential benefits of increased spatial resolution in DAA become apparent as finer control point distributions are implemented. The increased resolution allows for a broader capture of morphological diversity, including anatomical features such as horns, which are often underrepresented in manual landmarking approaches. However, this improvement can also introduce greater variability in results, potentially reducing phylogenetic signal and altering interpretations compared to traditional landmark-based analyses [60]. This reflects that landmark-free methods such as DAA differ substantially from manual landmarking in their application, so these differences should be considered when comparing results.

At present, landmark-free methods like DAA are still evolving, and their applicability should be carefully considered, particularly when integrating datasets with mixed modalities that cannot be easily standardised, or when dealing with specimens that have missing or incomplete anatomical structures. Nevertheless, with the advancements made in this study and previous research [24, 39], along with the proposed next steps outlined earlier, we believe landmark-free approaches offer a promising avenue for achieving high-resolution spatial comparisons across taxa, even in cases where homologous points are scarce or difficult to define.

## Conclusions

Landmark-free methods such as DAA represent a powerful and innovative approach to morphometric analyses, particularly for large-scale comparative studies. By eliminating the need for manual landmark placement, these methods enhance efficiency, reduce user bias, and allow for higher spatial resolution in shape comparisons. Our results demonstrate that DAA successfully captures biologically meaningful patterns while maintaining congruence with manual landmarking approaches.

Although some reductions in phylogenetic signal and variations in shape space stability were observed with differing control point densities, these challenges provide opportunities for refinement rather than fundamental limitations. Continued advancements in mesh parcellation, automated segmentation, and computer vision techniques for imputing missing anatomical regions and filtering non-homologous features (e.g., horns, teeth) are likely to further strengthen the reliability of landmark-free methods. These developments will improve the ability of DAA to preserve homology and handle incomplete specimens, making it an increasingly viable alternative to traditional approaches.

Given its scalability and potential for high-resolution shape analyses across taxa with few shared homologous points, DAA and similar landmark-free methods are poised to become an integral part of the morphometric toolkit. As the field progresses, integrating these approaches with existing methods may unlock new insights into morphological evolution while maintaining the rigor and biological validity of shape analyses.

## Materials and methods

We obtained .ply meshes and geometric morphometric data for manually collected landmarks and semilandmarks from Goswami et al. [40]. This dataset comprised 322 crown and stem placental mammals, including 207 extant and 115 extinct species (Additional file, data A20).

The manual landmarking scheme consisted of 66 three-dimensional (3D) landmarks and 69 semilandmark curves collected for the left side of the skull, utilising Stratovan Checkpoint (Stratovan, Davis, CA, USA). Landmarks and semilandmarks were imported into R v.4.3.1 for analysis, where curves were resampled to a common number of semilandmarks and slid to minimise bending energy using Morpho [46] v.2.11, which measures and optimises local shape differences versus the mean shape. Generalised Procrustes analysis in geomorph [47] v.4.05 was then used to register the landmarks, resulting in a total of 754 3D landmarks and sliding semilandmarks (Additional file, Data A21-22). Principal component analysis was performed using Procrustes-aligned 3D data in R.

### Mesh processing

To facilitate a direct comparison between the manual landmarking data and the Deterministic Atlas Analysis (DAA) method, it is essential to remove any effects of translation, rotation, and scaling. To achieve this, we applied a Procrustes transformation to standardise all 322 meshes, using the *rotmesh.onto* function in Morpho [46] v.2.12. This function aligns each mesh to a target landmark configuration by applying a rigid-body transformation (translation and rotation). When scaling is enabled, it also performs a scaling adjustment based on generalised Procrustes analysis (GPA)**,** minimising the least-squares differences between corresponding input and reference landmarks according to the ratio of their centroid sizes. In our analysis, all data were mapped onto the coordinates of the first mesh in our dataset specifically that of *Acinonyx jubatus*, using the mirrored manual landmark data, which had not undergone GPA-based scaling, as the reference (Additional File, Data A22). This approach ensured the dataset was free of size-related effects, allowing for direct comparisons of shape variation.

The meshes in the selected dataset were produced using a combination of computed tomography (CT) and surface scanning techniques (see Additional File, data A20), resulting in the varied properties among the meshes. Notably, the CT-derived meshes have open surfaces (Fig. 1a), while surface scan data is characterised by closed surfaces. Previous studies have indicated that these differences in topology can significantly influence the outcomes of landmark-free methods [48, 49]. Thus, we aimed to investigate the impact of utilising mixed modalities when comparing shapes through the DAA method. To address potential effects stemming from the presence of both open and closed surfaces we created two distinct input datasets: (1) “Aligned-only” meshes, which comprise the original aligned meshes with both open and closed surfaces, and (2) “Poisson” meshes, which consist exclusively of watertight closed meshes.

To generate these watertight meshes, we used voxelisation and segmentation in Dragonfly v.2021.3 (Object Research Systems, Canada) to initially close any open holes. Subsequently, to ensure that the meshes were watertight and to evenly redistribute the faces and vertices across the mesh, we applied a Screened Poisson distribution [50] in Meshlab v.2023.12 [74]. The Poisson reconstruction algorithm focuses on the reconstruction of smooth surfaces while approximating the underlying point clouds, as described by the equation [50]:

$${\Delta }_{\upchi }\equiv \nabla \cdot {\nabla }_{\upchi } =\nabla .\text{V}.$$

Where $\Delta\upchi$ is the Laplacian of the scalar function, which defines the implicit surface to be reconstructed, $\nabla \cdot$ denotes the divergence operator and $\text{V}$ is a vector field derived from the normals of the point cloud data. The purpose of the $\Delta\upchi$ function is to preserve the topology and geometric properties of the mesh by aligning it with that of $\text{V}$.

Once meshes for both input datasets for DAA were processed, we decimated the “Aligned-only” and “Poisson” meshes to 50,000 faces using a quadratic mesh decimation approach using trimesh v.3.19.4 in Python v.3.8.8. This decimation was conducted to reduce computational demand while maintaining the overall geometric topology.

### Deterministic Atlas Analysis (DAA)

Deterministic Atlas Analysis (DAA) is a method based on large deformation diffeomorphic metric mapping (LDDMM)**,** which compares shapes by applying diffeomorphic transformations within 2D or 3D ambient space. DAA is unique in that it learns a template shape which corresponds to the average of the selected dataset [33], in this case the 322 selected specimens. However, before estimating this mean shape, the method requires the selection of an initial template from within the dataset. Given that previous studies suggest a single template may bias results [51–53], we tested three different specimens as the template chosen based on the manual landmarking results. Specifically, we selected *Arctictis binturong* to represent a shape close to the mean, *Cacajao calvus* as an intermediate form, and *Schizodelphis morckhoviensis* as an extreme shape.

For each initial template, DAA automatically estimates a new mean shape that represents the average morphology of the 322 specimens. This process relies on geodesic regression, a method that operates within a Riemannian manifold [36, 37], enabling the generalisation of shape differences across curved surfaces in higher-dimensional space. For the geodesic regression model that best fits the collection of meshes ${{(C}_{i})}_{i=1,\dots ,n}$ observed at times ${{(t}_{i})}_{i=1,\dots ,n}$, the alignment is optimised by minimising a loss function, which is mathematically defined as [34]:

$${\text{f}\left(T,q,({\mu }_{i}\right)}_{i=1,\dots ,n})= \sum_{i=1}^{n}\left(d\left({\Phi }_{q,{\mu }_{i} }\left(T\right), {C}_{i}\right)\right)+R(q, {\mu }_{i}))$$

Where ${\text{f}\left(T,q,({\mu }_{i}\right)}_{i=1,\dots ,n})$ represents the total cost of aligning template $T$ to all obseved specimen shapes ${C}_{i}$ across the dataset, with the summation performed over all specimens $n$. The term $\left(d\left({\Phi }_{q,{\mu }_{i} }\left(T\right), {C}_{i}\right)\right)$ quantifies the deformation distance between the transformed template ${\Phi }_{q,{\mu }_{i}}$ and the observes shape ${C}_{i}$. This transformation is parameterised by the momenta $q$ and the mean shape estimate ${\mu }_{i}$, which collectively describe the optimal deformation trajectory. The term $R(q,{\mu }_{i}))$ serves as the regularisation function, allowing smoothness in the deformations and preventing overfitting to local variations. Through this testing, we found that *A. binturong*, the shape closest to the mean in the manual landmarking analysis was the best performing initial template. Subsequently, we tested a range of Gaussian kernel widths: 40.0 mm, 20.0 mm, and 10.0 mm, to assess the influence of this parameter on capturing shape variation. The Gaussian kernel width determines the spatial extent of the diffeomorphic comparisons through a smoothness constraint. This spatial extent is controlled by generating control points *p*, which serve as references for shape comparisons, effectively replacing traditional landmarks. Within this framework, the deformations for a set of *n* control points can be defined as follows [34]:

$$X\left(x\right)= \sum_{i=1}^{p}K \left(x, {q}_{i}\right)\cdot {\mu }_{i}$$

Where ${{(q}_{i})}_{i=1,\dots ,n}$ represents the control points, each associated with the momentum vectors (momenta) ${{(\mu }_{i})}_{i=1,\dots ,n}$ that generate a vector field $X$ within 3D space. Specifically, the term $X\left(x\right)$ described the vector field at position $x$. Here, the size of the kernel width is inversely related to the number of control points, meaning a larger kernel width results in fewer control points. The spatial extent of these points is mathematically defined through a Gaussian kernel as follows [34] :

$${K}_{\sigma }\left(\text{x},\text{y}\right)=\text{exp}\left(-\frac{{\parallel \text{x}-\text{y}\parallel }^{2}}{{\sigma }^{2}}\right)$$

Where *x* and *y* represent points in space, σ is the kernel width (or scale), which determines the extent of the interaction between the points, and ∥*x*−*y*∥ is the Euclidean distance between *x* and *y*.

Within this framework, a time dynamic is prescribed to the control points and the momenta, which correspond to the geodesic equation considered in the ambient space. Within Hamiltonian equations these are described as [34]:

$$\left\{\begin{array}{c}\dot{q}\left(t\right)=K[q\left(t\right), q\left(t\right)]\cdot \mu (t)\\ \dot{\mu }\left(t\right)=- \frac{1}{2}{\nabla }_{q}\left\{K[q\left(t\right), q\left(t\right)]\cdot {\mu (t)}^{T}\mu (t)\right\}\end{array}\right.$$

Within this system these equations are solved using the numerical integration methods of Euler and Runge-Kutta, to approximate the trajectory of the control points and momenta over time. Each iteration of the data involves computing convolutions between the control points and momenta within a quadratic form to update the system dynamics.

When using meshes, deformations can be described through their vertices ${({v}_{p})}_{p=1, \dots ,d}$ and their connectivity. For each mesh it is possible to compute the centres ${({c}_{p})}_{p=1, \dots ,f}$ and normals of the faces or edges ${({n}_{p})}_{p=1, \dots ,f}$. In this context, two types of distances are considered to quantify the differences between meshes. The first measures the current pointwise distance between corresponding points of two meshes defined as:

$$d\left({\left(n_p^\alpha,c_p^\alpha\right)}_{p=1,\dots,f^\alpha}, {\left(n_q^\beta,c_q^\beta\right)}_{q=1,\dots,f^\beta}\right)^2=\sum\limits_p {\sum\limits_q K^W}\left(c_p^\alpha,c_q^\beta\right)\left(\left(n_p^\alpha\right)^{\top} n_q^\beta\right).$$

Where ${K}^{W}$ is the gaussian kernel with the width ${\sigma }^{W}$. The second measure is the varifold distances between meshes which can be described as:

$$d\left({\left(n_p^\alpha,c_p^\alpha\right)}_{p=1,\dots,f^\alpha}, {\left(n_q^\beta,c_q^\beta\right)}_{q=1,\dots,f^\beta}\right)^2= \sum\limits_p \sum\limits_q K^W\left(c_p^\alpha,c_q^\beta\right)\frac{\left[\left(n_p^\alpha\right)^{\top} n_q^\beta\right]^2}{\left\|n_p^\alpha \parallel n_q^\beta\right\|}.$$

Where ${K}^{W}$ is the gaussian kernel with the width ${\sigma }^{W}.$ Based on this mathematical framework, we executed the analyses using *Deformetrica 4* [33, 34]. All analyses were executed for 150 iterations using an initial step size of 0.01 and with the noise parameter set to 10.0.

### Kernel principal component analysis (kPCA)

The DAA produces three key outputs: (1) the initial position of the control points in relation to the atlas, (2) the momenta for each of the 322 specimens that represent the measured deformations and (3) the final atlas template. To be able to compare the shape variation, we focus on comparing the values of the momenta. The initial properties of these momenta are non-linear due to the use of a geodesic on a non-linear manifold of diffeomorphisms [32]. Thus, to be able to compare these values, the results must be projected into linear space. To do this we use non-linear kernel principal component analysis (kPCA) [38]. This technique differs from traditional PCA and is more appropriate here as it can handle non-linear data. It can do this by projecting the momenta into a higher-dimensional space, which then makes the data linearly separable. This is done by calculating the dot product of the mapped data points [38]:

$$\text{K}\left({x}_{i},{x}_{j}\right)=\langle \phi \left({x}_{i}\right). \phi \left({x}_{j}\right)\rangle$$

Which is projected into the principal component space:

$$\left({V}^{k}. \phi \left(x\right)\right)= \sum_{i=1}^{N}{\alpha }_{i}^{k}(\phi \left({x}_{i}\right). \phi (x)).$$

Where ${V}^{k}$ is the $k$ -th eigenvector in the feature space and ${\alpha }_{i}^{k}$ are the components of the $k$ -th eigenvector from the kernel matrix decomposition.

When using a manual landmarking approach, the number of PC axes is defined by the number of specimens (n − 1), in this case 321 axes. As a result, for the DAA analyses, we opted to project the measured momenta into 321 PC axes to align with the results from the manual landmarking. For the kPCA, each axes represents the maximum variance of the kernel matrix as opposed to the original space as is the case with traditional PCA. Each axis is still ordered in relation to the amount of variation described for the data. This projection into 321 axes was conducted using 1000 iterations and a kernel gamma of 2.5 × 10^−6^, where the gamma value determines the width (or smoothness) of the kernel.

All analyses (DAA and kPCA) were executed on a workstation running Ubuntu (22.04.2) for Windows, equipped with an Intel(R) Core (TM) i7-7700 CPU operating at 3.60 GHz, 64.0 GB of RAM, and an NVIDIA GeForce RTX 2090 graphics card with 8.0 GB of dedicated GPU memory.

### Comparing the manual landmarking and landmark-free methods

To compare the results obtained from manual landmarking with those generated by each DAA, we assessed a variety of statistical measures. First, to estimate the correlation in shape measurements between individuals estimated using the two distinct methods and across kernel widths, we calculated the Euclidean distances from each specimen to the atlas specimen, *A. binturong*. This was done across all 321 axes for both the manual landmarking and each DAA. This distance is defined as [41]:

$$d\left(p, q\right) = \sqrt{\sum_{i=1}^{n} \left(q_i - p_i\right)^2}$$

Where, *p, q* are two points in Euclidean n-space,${q}_{i } {p}_{i}$ are Euclidean vectors, starting from the origin of space (initial point). We then compared the distances derived from the manual landmarking with those from the DAA, employing bivariate plots and assessing their correlations using linear regression models in R v.4.3.1. This analysis allowed us to quantify the overall correlations across the entire dataset.

To further evaluate the correlation between the two methods, we measured the overall correspondence between the distance matrices, using two complementary approaches. First, we applied a Mantel test [42, 43] to assess the statistical correlation between the matrices. This works by measuring the association of pairwise distances between each of the selected matrices. Second, we used the PROcrustean Randomisation TEST (PROTEST) [44, 45] to directly quantify the degree of overlap between the matrices. This test evaluates the significance of the Procrustes fit between two different configurations. Both analyses were performed using vegan [75] v.2.6 in R. We applied both techniques across all 321 axes to assess the overall correlation between datasets, conducting each across 9999 permutations.

We also measured the correlation between the percentage of variation (eigenvalues) explained by each of 321 axes for the manual landmarking and DAA, using linear regression models to determine the overall correlation and statistical significance of each result.

### Comparison of methods using heatmaps

To examine how the estimates of shape variation correlate with the measurements obtained directly from each cranium, we generated heatmaps for all eight orders that contained more than ten specimens in the analysis (Additional File, Figure A7). Generating these heatmaps relied on comparing each of the selected specimens with the estimated mean shapes from both methods.

For the manual landmarking approach, we first mirrored the GPA-aligned manual landmark data to ensure coverage on both sides of the crania. Using these mirrored landmarks, we calculated the average position of each landmark across the 322 specimens to create a mean landmark configuration. For each of the eight selected specimens, we then warped the mesh to this mean configuration, using the specimen-specific manual landmarks as the reference matrix to quantify the deformation. This transformation was performed using a thin-plate spline interpolation via the *tps3d* function in Morpho [46]. The *meshDist* function, was then used to measure the Euclidean distances of the corresponding vertices between the original and warped meshes.

For the DAA method, the mean shape of the population is automatically estimated during atlas construction using the geodesic regression function and is provided directly as an output [33]. Consequently, a separate morphing procedure based on control points is not required. Since the mean shape varies depending on the selected kernel width, we compared each of the eight specimens with the mean shape predicted under each analysis. To do this, we used the *meshDist* function in Morpho [46] to calculate the Euclidean distances between corresponding vertices of each specimen and the predicted mean atlas shape. Heatmaps were used to visualise the magnitude of the Euclidean distances.

### Comparisons of downstream macroevolutionary analyses

We assessed the impact of using each of the different methodologies on the downstream macroevolutionary analyses. First, we randomly selected a phylogenetic tree as used in Goswami et al. [40] to pair with the results of the morphometric analyses. Using geomorph [47] v.4.05 in R, we calculated and compared the phylogenetic signal for each analysis through the *physignal* function, applying the K_mult_ statistic [54] through 99 iterations to test the fit of the data with the phylogeny. Next, we measured and compared the morphological disparity and evolutionary rates associated with different dietary categories and locomotor types (the only complete categories for all of the 322 specimens), employing the *morphol.disparity* and *compare.evol.rates* functions in geomorph [47] v.4.05, respectively. For the evolutionary rates estimate, we used a simulation-based method, running each analysis through 100 iterations to ensure robust comparisons. We compared the values of morphological disparity and evolutionary rates for the manual landmarking and DAA using Spearman’s rank correlation coefficient and producing bivariate plots in R.

## Supplementary Information

Supplementary Material 1.

## Data Availability

All the final data used in the paper are freely available in the Supplementary Material and at https://github.com/JamesMulqueeney/Deterministic-Atlas-Analysis. All the code used in the paper is also stored in this GitHub repository. All 3D meshes for each specimen are available for free download on phenome10k.org or morphosource.org. Each of the corresponding accession numbers and accessibility details are found in the supplementary material file, Data_A20-Specimen_Details.csv, which can be found in the Supplementary Material or GitHub page. Processed data and other large files are freely available in the following DOI: 10.5258/SOTON/D3451. Extensive descriptions on the methodology and functions can be found at https://gitlab.com/icm-institute/aramislab/deformetrica/-/wikis/home and the full descriptions of the pipeline can be found at https://gitlab.com/ntoussaint/landmark-free-morphometry.
