# Supplementary material for: Assessing the application of landmark-free morphometrics to macroevolutionary analyses
Source: BMC Ecol Evol. 2025 Apr 27;25:38. doi: 10.1186/s12862-025-02377-9 (PMC12034209; doi:10.1186/s12862-025-02377-9)
Supplement: Supplementary file 1 — Supplementary Material 1. [file 12862_2025_2377_MOESM1_ESM.zip › Supplementary Material/Automated Method Comparison Paper_BMC_Supplementary.docx]

**Assessing the application of landmark-free morphometrics to macroevolutionary analyses**

James M. Mulqueeney^1, 2^, Thomas H. G. Ezard^1^ & Anjali Goswami^2^

^1^School for Ocean & Earth Science, National Oceanography Centre Southampton, University of Southampton Waterfront Campus, Southampton, UK

^2^Department of Life Sciences, Natural History Museum, London, UK

Corresponding Author: James M. Mulqueeney (j.m.mulqueeney@soton.ac.uk)

**Additional Files**

**Tables**

**Table A1.** Correlations measures (R^2^) of pairwise Euclidean distances between the manual landmarking and Deterministic Atlas Analysis (DAA) across each of the different kernel widths. All values are significant.

| **Mesh Type** | **Kernel 40** | **Kernel 20** | **Kernel 10** |
| --- | --- | --- | --- |
| Aligned-Only | 0.147 | 0.126 | 0.171 |
| Poisson | 0.532 | 0.434 | 0.425 |

**Table A2.** Measurements of correlation using Spearman’s rank correlation coefficient between the measures of morphological disparity and evolutionary rates calculated using the *morphol.disparity* and *compare.evol.rates* functions in geomorph [1] v.4.05. Results are given for comparing the manual landmarking approach with each of Deterministic Atlas Analysis (DAA) using a kernel width of 40.0mm, 20.0mm and 10.0mm for groupings based on diet and locomotion. All significant values are highlighted in bold (p <0.05).

| **Dataset** | **Diet Disparity** | **Diet Rate** | **Locomotion Disparity** | **Locomotion Rate** |
| --- | --- | --- | --- | --- |
| Poisson 40 | 0.429 | **0.857** | **0.800** | **0.933** |
| Poisson 20 | 0.321 | **0.679** | **0.850** | **0.933** |
| Poisson 10 | 0.321 | **0.929** | **0.733** | **0.883** |

**Figures**


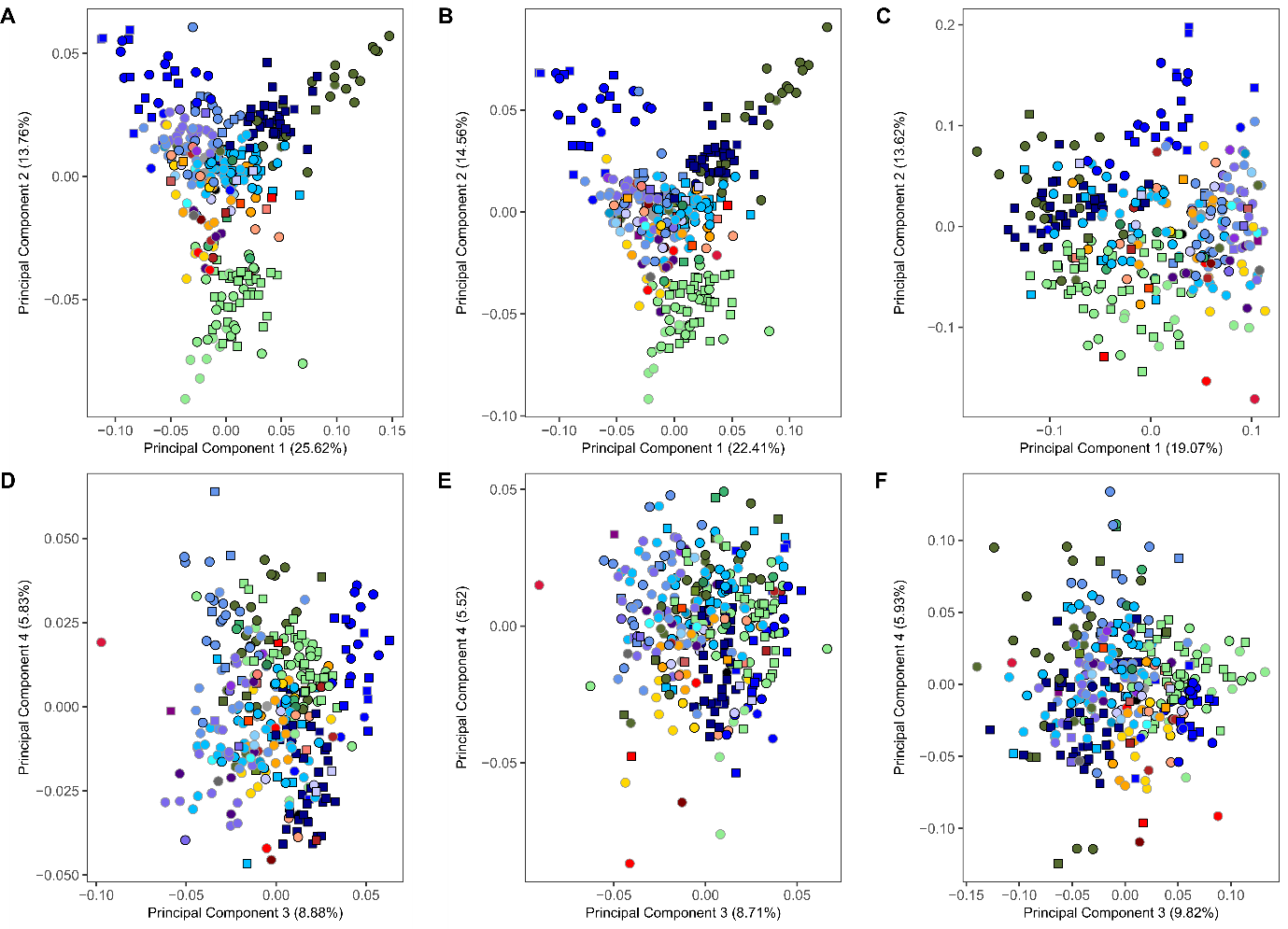


**Figure A1.** Principal component (PC) plots for PC axes 1 to 4 generated using a kernel width of 20.0 mm to compare different atlases. These include (a, d) Arctictis binturong (270 control points), (b, e) Cacajao calvus (420 control points), and (c, f) Schizodelphis morckhoviensis (32 control points). The first two principal components (PC1 and PC2) are shown in (a–c), while PC3 and PC4 are shown in (d–f). The results indicate that atlas selection influences the number of control points in conjunction with the kernel width parameter. Most shape measurement differences arise from variations in the number of control points per specimen, however, using an atlas that deviates significantly from the mean can also lead to misplacement of certain specimens toward the centre of the PC plot.


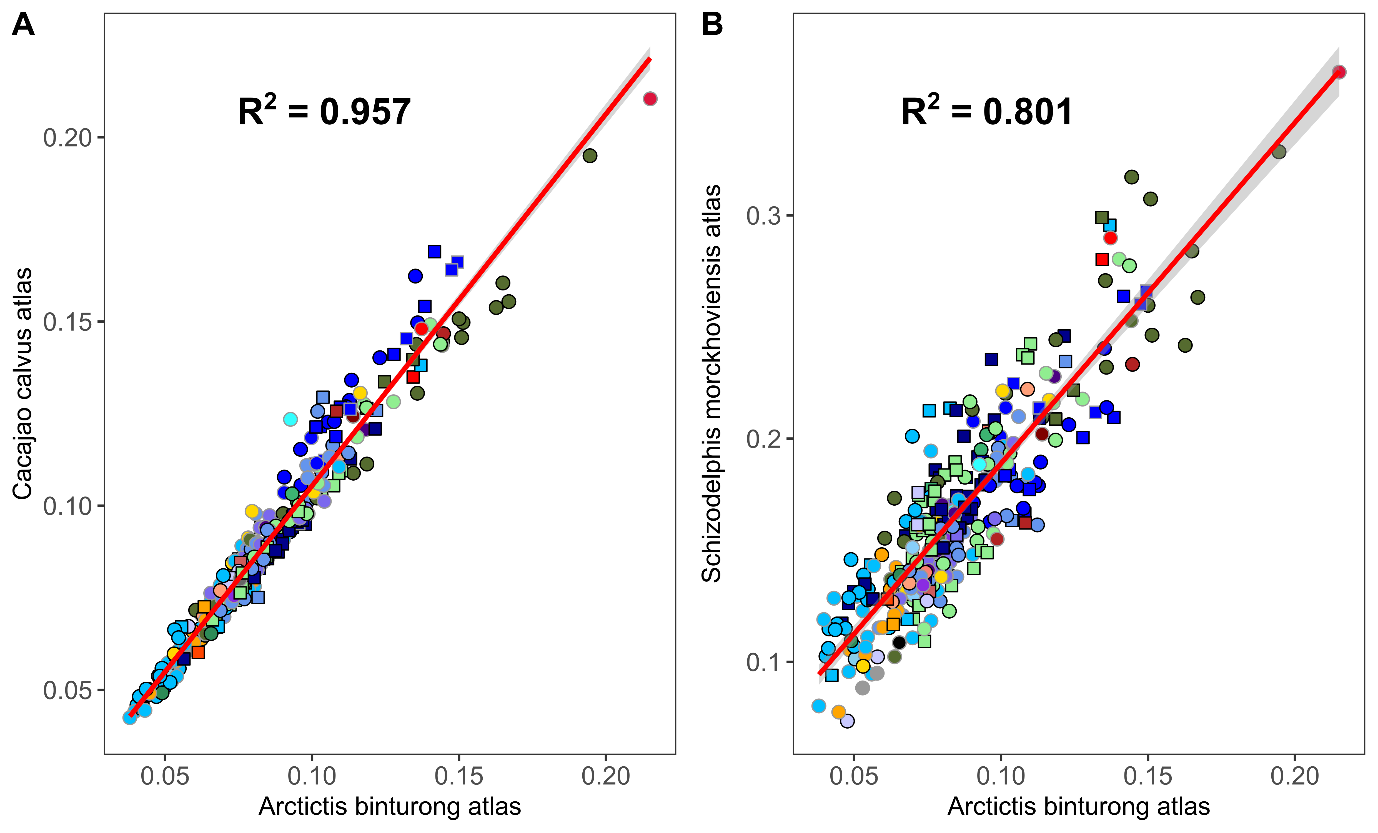


**Figure A2.** Pairwise Euclidean distance comparisons between each specimen to *Arctictis binturong* used to compare the correlation between the results obtained using a kernel width of 20.0mm for an atlas of *A. binturong* resulting in 270 control points with using an atlas of (a) *Cacajoa calvus* which results in 420 control points and (b) *Schizodelphis morckhoviensis* atlas resulting in 32 control points. The results show that the selection of atlas does not directly affect the shape measurements but does have a significant impact on the number of control points that are generated alongside the kernel width parameter. We still recommend the use of an atlas close to the mean shape (which can be estimated using the landmarks used for alignment) to obtain the best results.

**
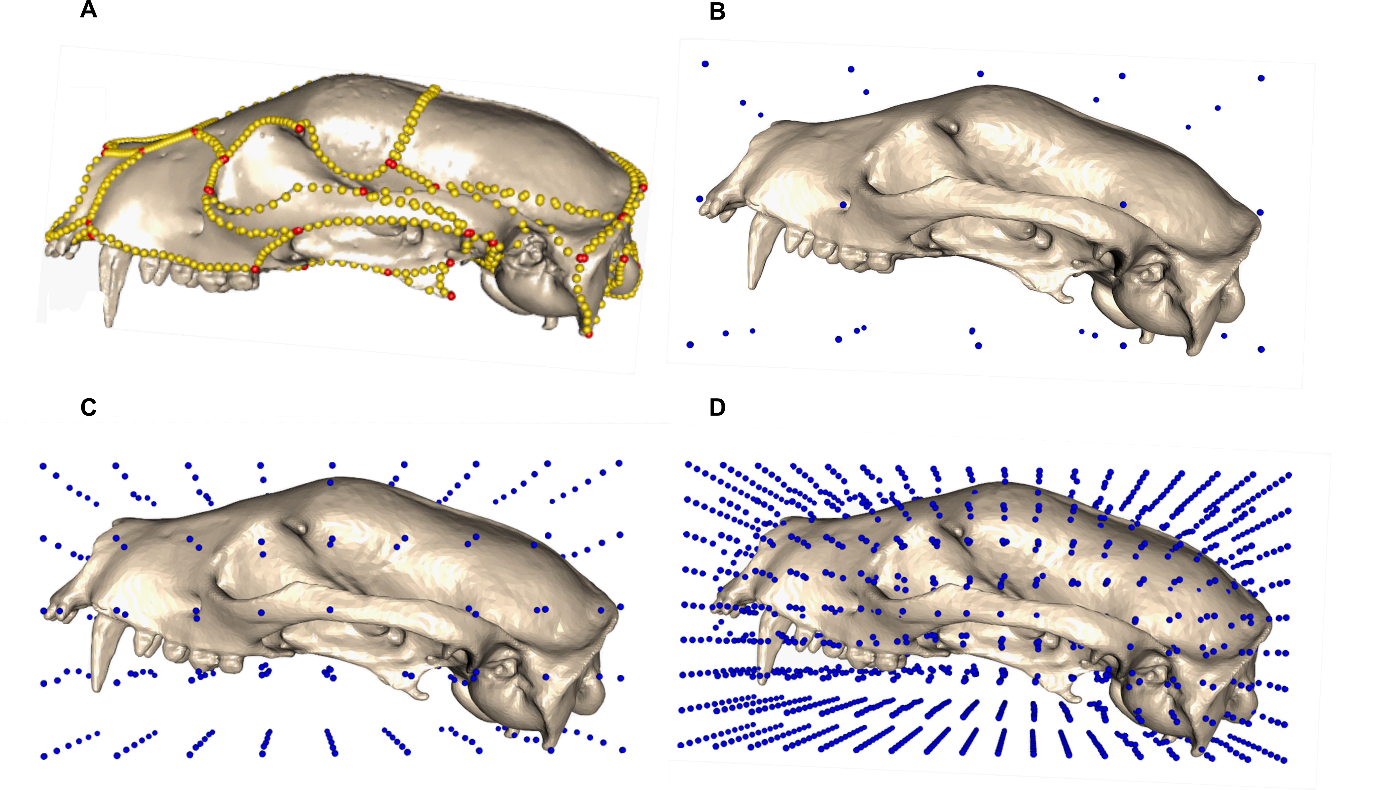
**

**Figure A3.** Comparative assessment of morphometric methods applied to the 3D mesh of the atlas specimen Arctictis binturong (MNHN 1936-1529). The figure contrasts (a) a manual landmarking approach using 754 landmarks and sliding semilandmarks with unmapped control points obtained through Deterministic Atlas Analysis (DAA) under different kernel widths: (b) 40.0 mm, producing 45 control points; (c) 20.0 mm, producing 270 control points; and (d) 10.0 mm, producing in 1,782 control points.

**
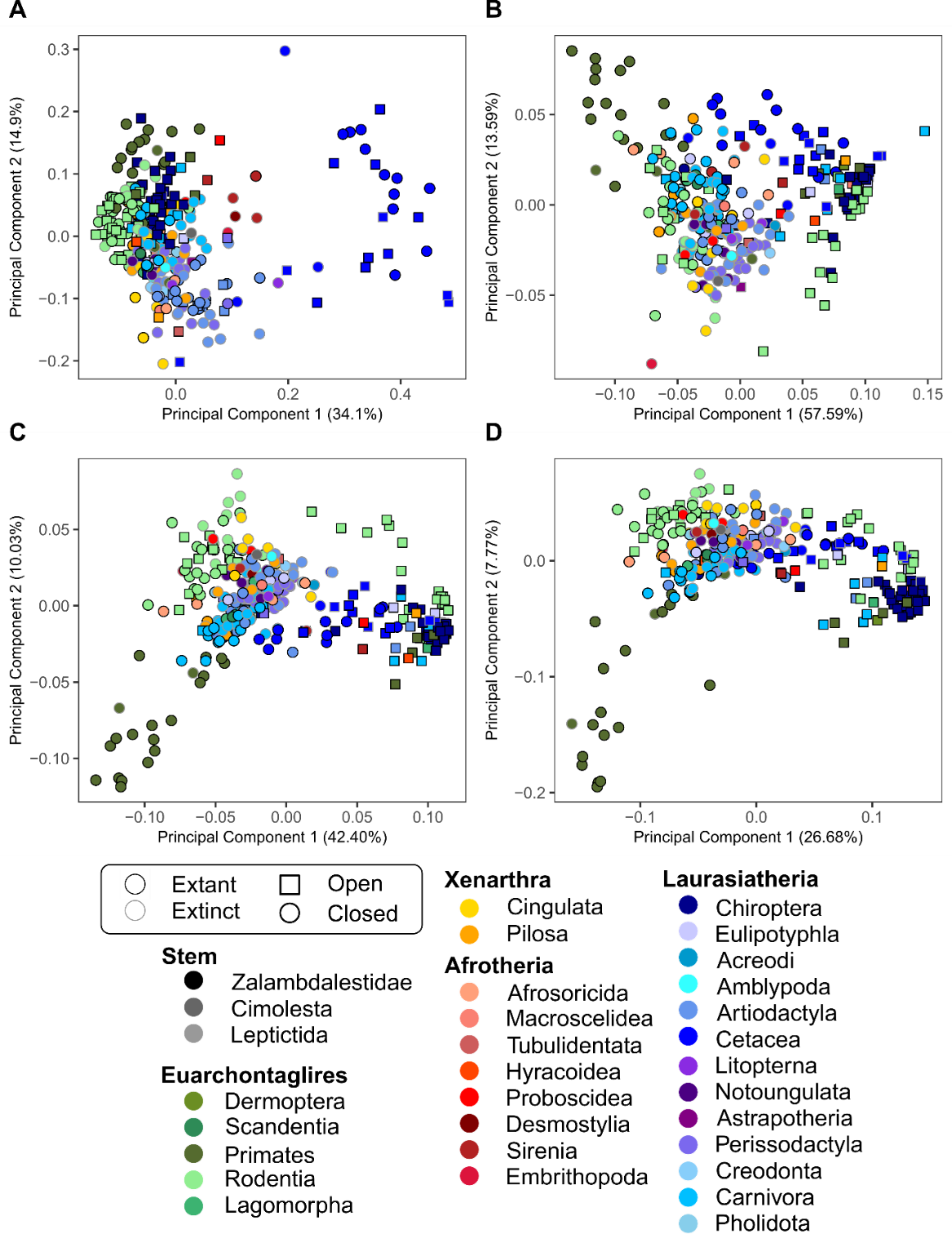
**

**Figure A4.** Principal component plots (PC) for PC1 and PC2 obtained for the shape analysis using the “Aligned-only” meshes, comparing the results between (a) manual landmarking with 754 landmarks and sliding semilandmarks and the DAA method using kernel widths of (b) 40.0 mm, yielding 45 control points, (c) 20.0 mm, yielding 270 control points, and (d) 10.0 mm, yielding 1,782 control points. The results highlight the issue of using mixed modalities (open and closed surfaces) without standardisation in the DAA.


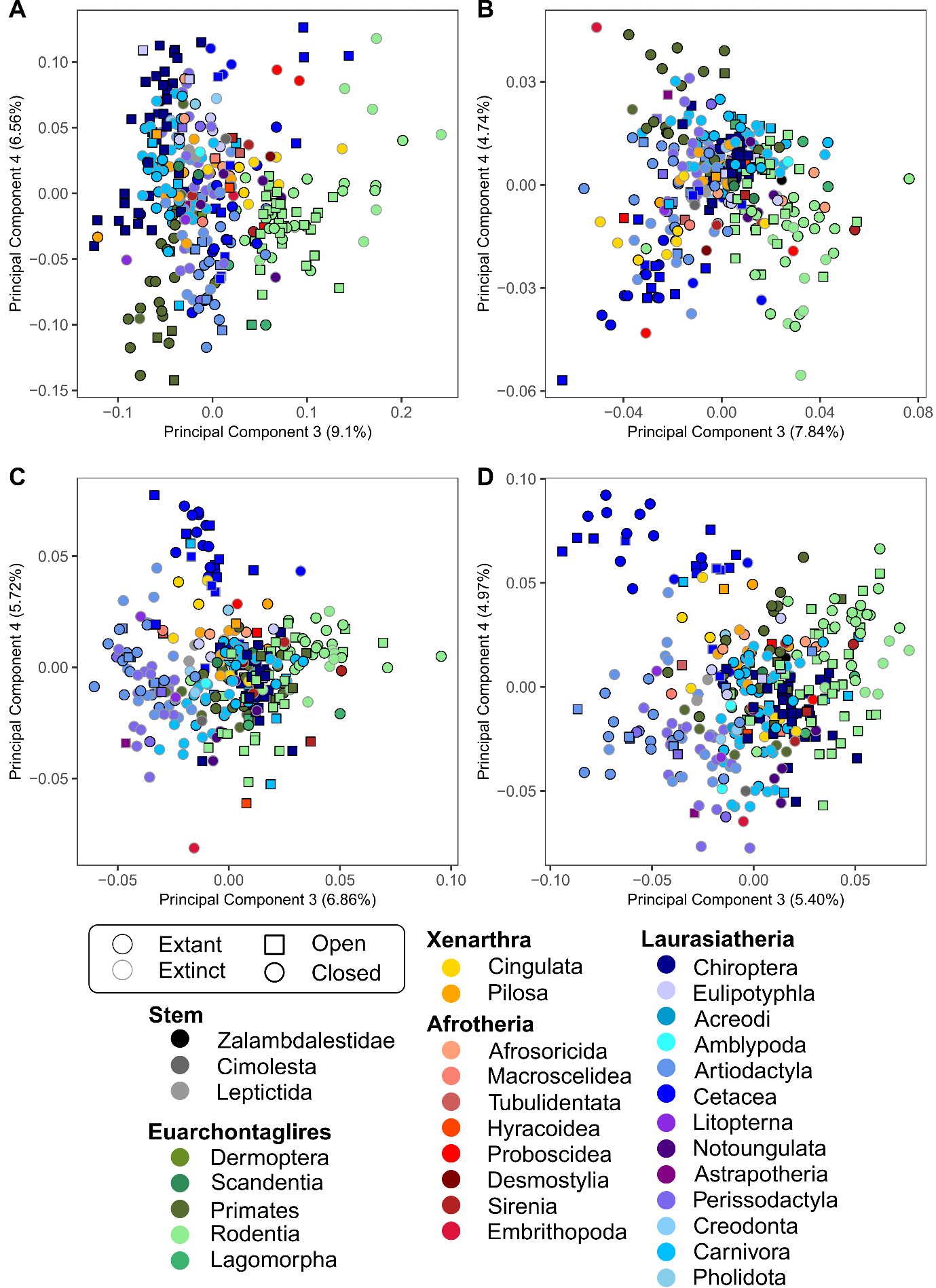


**Figure A5.** Principal component plots (PC) for PC3 and PC4 obtained for the shape analysis using the “Aligned-only” meshes, comparing the results between (a) manual landmarking with 754 landmarks and sliding semilandmarks and the DAA method using kernel widths of (b) 40.0 mm, yielding 45 control points, (c) 20.0 mm, yielding 270 control points, and (d) 10.0 mm, yielding 1,782 control points. The results highlight the issue of using mixed modalities (open and closed surfaces) without standardisation in the DAA.

**
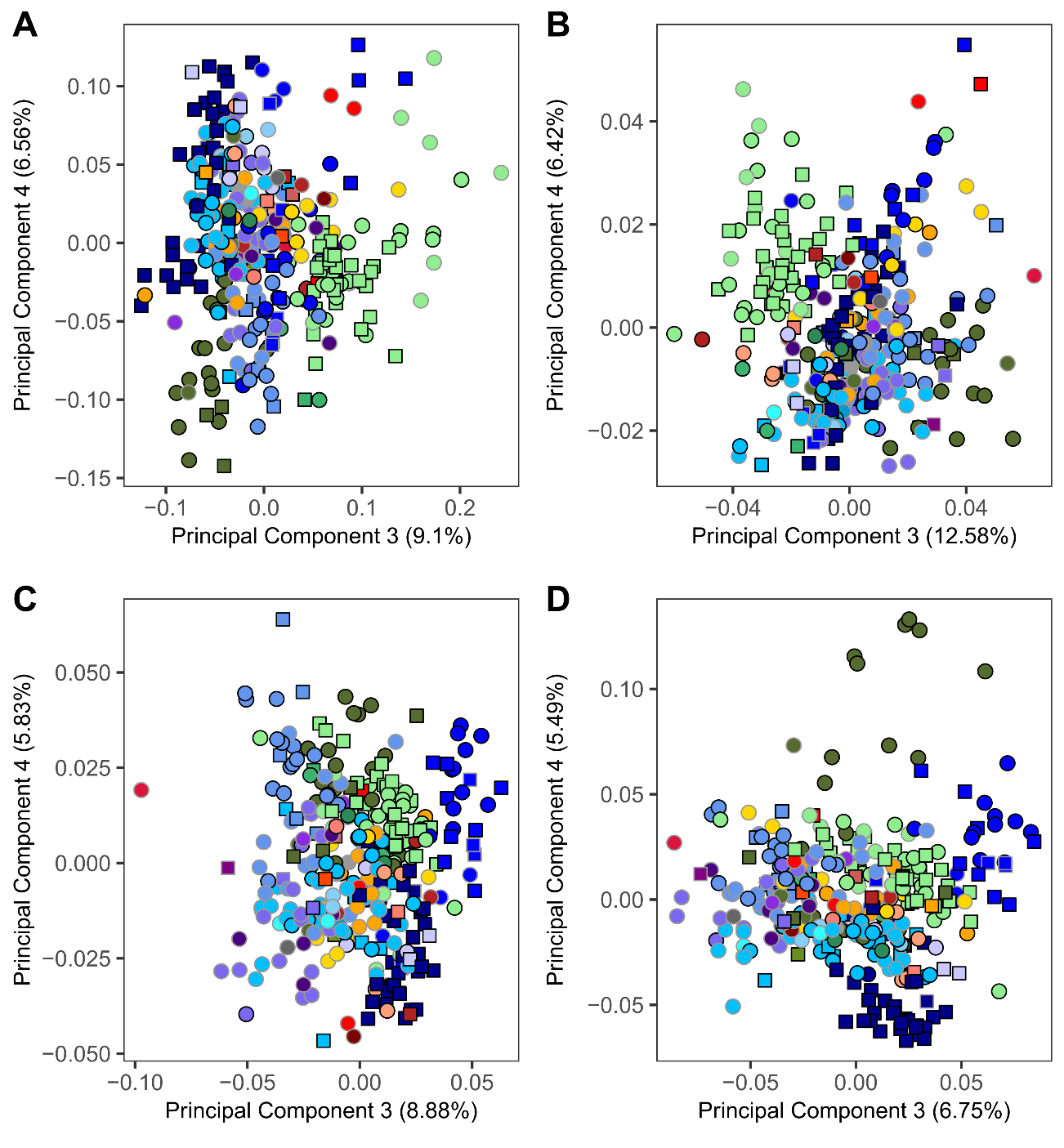
**

**Figure A6.** Principal component plots (PC) for PC3 and PC4 obtained for the shape analysis using the “Poisson” meshes, comparing the results between (a) manual landmarking with 754 landmarks and sliding semilandmarks and the DAA method using kernel widths of (b) 40.0 mm, yielding 45 control points, (c) 20.0 mm, yielding 270 control points, and (d) 10.0 mm, yielding 1,782 control points. The results highlight how generating Poisson meshes aid in removing the effect of modalities.


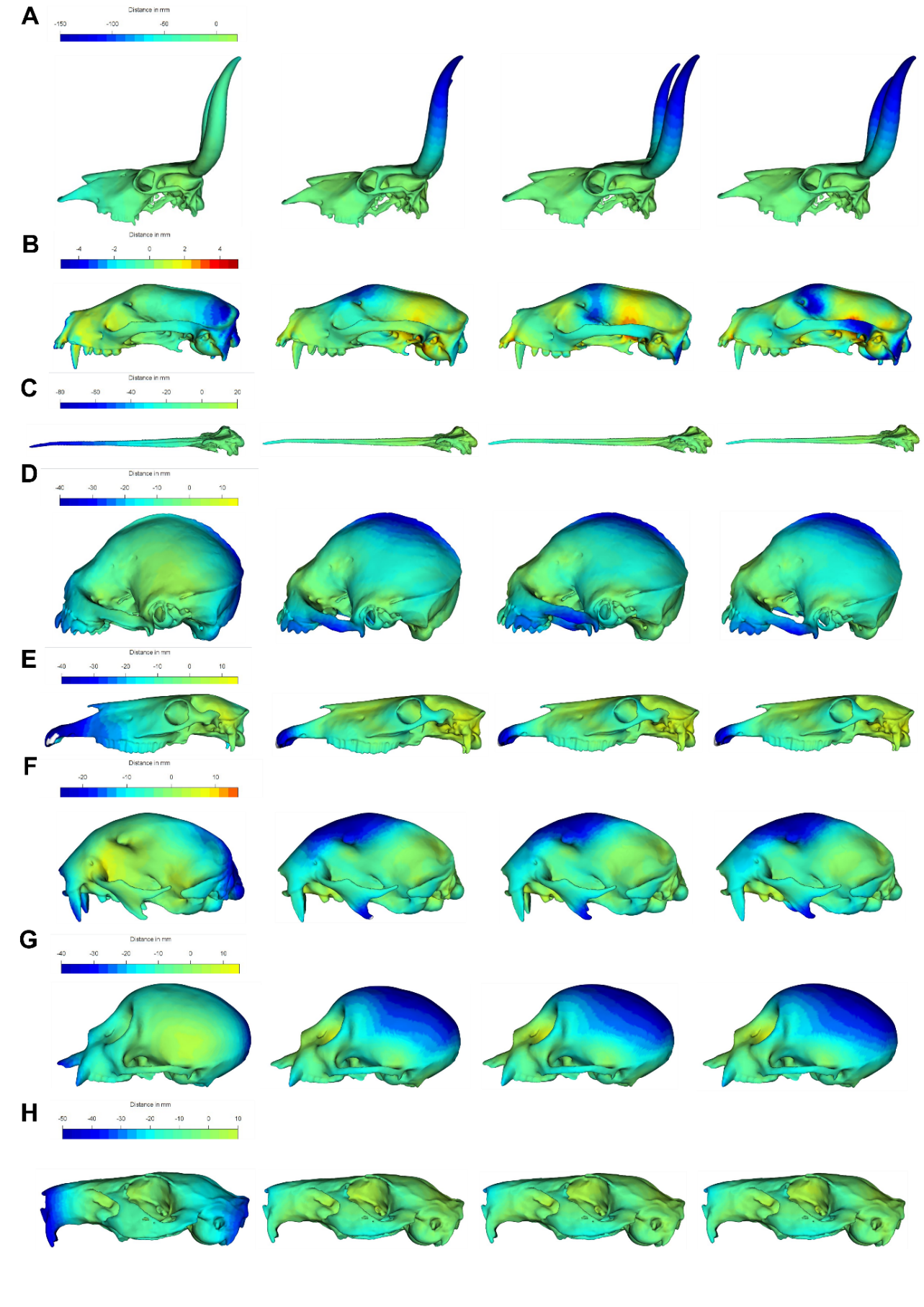


**Figure A7.** Displacement heatmaps for (a) *Bos taurus* (NHMUK ZD 1981.984), (b) *Arctictis binturong* (MNHN-ZO-AC 1936-1529), (c) *Schizodelphis morckhoviensis* (USNM 13873), (d) *Centurio senex* (AMNH, M-249109), (e) *Equus caballus* (LDUCZ z.3062), (f) *Choloepus hoffmanni* (AMNH M-30765), (g) Cacajao calvus (NHMUK ZD 1928.4.27.6) and (h) *Cavia aperea* (NHMUK ZD 1901.11.3.78). From left to right, the figure shows heatmaps for comparing the Euclidean distances between each specimen and the estimated mean shape from the manual landmarking scheme with 754 landmarks and sliding semilandmarks, and the geodesic mean shape generated via Deterministic Atlas Analysis (DAA) for a kernel width of 40.0 mm with 45 control points, a kernel width of 20.0 mm with 270 control points, and a kernel width of 10.0 mm with 1,782 control points. The heatmaps demonstrate the differences in where shape variation is captured for both methods.

**
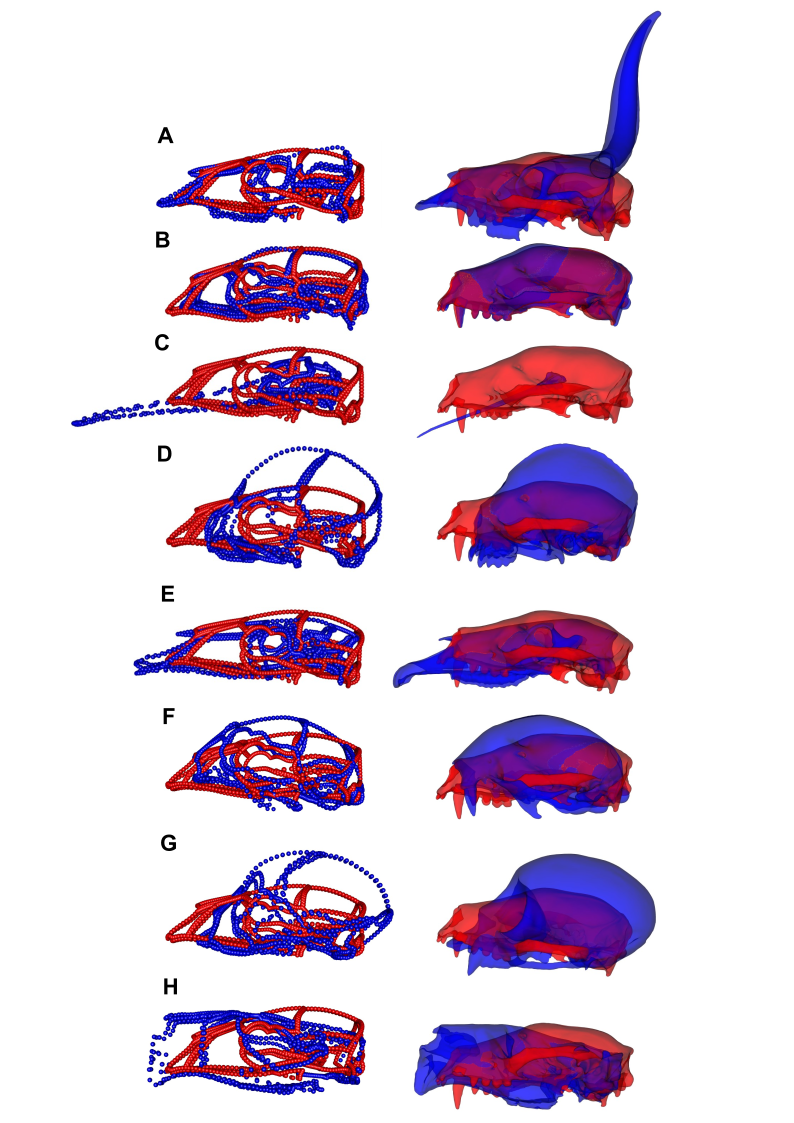
**

**Figure A8.** Comparison of generalised Procrustes analysis (GPA) scaling using (left) manual landmarks and (right) *rotmesh.onto* function in Morpho [46] v.2.12 for input into Deterministic Atlas Analysis (DAA) for (a) *Bos taurus* (NHMUK ZD 1981.984), (b) *Arctictis binturong* (MNHN-ZO-AC 1936-1529), (c) *Schizodelphis morckhoviensis* (USNM 13873), (d) *Centurio senex* (AMNH, M-249109), (e) *Equus caballus* (LDUCZ z.3062), (f) *Choloepus hoffmanni* (AMNH M-30765), (g) Cacajao calvus (NHMUK ZD 1928.4.27.6) and (h) *Cavia aperea* (NHMUK ZD 1901.11.3.78).

**Data**

**Additional Data 1.** Kernel principal coordinates for deterministic atlas analysis measured using the Poisson mesh data for the *Arctictis binturong* initial template using a kernel width of 20.0mm (270 control points).

**Additional Data 2.** Kernel principal coordinates for deterministic atlas analysis measured using the Poisson mesh data for the Cacajao calvus initial template using a kernel width of 20.0mm (420 control points).

**Additional Data 3.** Kernel principal coordinates for deterministic atlas analysis measured using the Poisson mesh data for the *Schizodelphis morckhoviensis* initial template using a kernel width of 20.0mm (32 control points).

**Additional Data 4.** Kernel principal coordinates for deterministic atlas analysis measured using the aligned-only data using a kernel width of 40.0mm (45 control points).

**Additional Data 5.** Kernel principal coordinates for deterministic atlas analysis measured using the aligned-only data using a kernel width of 20.0mm (270 control points).

**Additional Data 6.** Kernel principal coordinates for deterministic atlas analysis measured using the aligned-only data using a kernel width of 10.0mm (1782 control points).

**Additional Data 7.** Kernel principal coordinates for deterministic atlas analysis measured using the Poisson mesh data using a kernel width of 40.0mm (45 control points).

**Additional Data 8.** Kernel principal coordinates for deterministic atlas analysis measured using the Poisson mesh data using a kernel width of 20.0mm (270 control points).

**Additional Data 9.** Kernel principal coordinates for deterministic atlas analysis measured using the Poisson mesh data using a kernel width of 10.0mm (1782 control points).

**Additional Data 10.** Correlation measures of Euclidean distance values within each major order (>10 specimens) across both the Aligned-only and Poisson mesh analyses.

**Additional Data 11.** Principal coordinates for shape data for the manual landmarking approach.

**Additional Data 12.** Eigenvalues generated for the kernel principal coordinate analysis for deterministic atlas analysis measured using the aligned-only data using a kernel width of 40.0mm (45 control points).

**Additional Data 13.** Eigenvalues generated for the kernel principal coordinate analysis for deterministic atlas analysis measured using the aligned-only data using a kernel width of 20.0mm (270 control points).

**Additional Data 14.** Eigenvalues generated for the kernel principal coordinate analysis for deterministic atlas analysis measured using the aligned-only data using a kernel width of 10.0mm (1782 control points).

**Additional Data 15.** Eigenvalues generated for the kernel principal coordinate analysis for deterministic atlas analysis measured using the Poisson mesh data using a kernel width of 40.0mm (45 control points).

**Additional Data 16.** Eigenvalues generated for the kernel principal coordinate analysis for deterministic atlas analysis measured using the Poisson mesh data using a kernel width of 20.0mm (270 control points).

**Additional Data 17.** Eigenvalues generated for the kernel principal coordinate analysis for deterministic atlas analysis measured using the Poisson mesh data using a kernel width of 10.0mm (1782 control points).

**Additional Data 18.** Estimated values of morphological disparity and evolutionary rates estimated for each different class of diet.

**Additional Data 19.** Estimated values of morphological disparity and evolutionary rates estimated for each different class of locomotion.

**Additional Data 20.** Specimen details and species trait data.

**Additional Data 21.** Generalised Procrustes analysis (GPA) shape data for the manual landmarking approach (not placed into a principal coordinate analysis).

**Additional Data 22.** Original landmark data for each species (mirrored for both sides of the cranium). These are used in the alignment of the meshes.

**Additional Data 23.** Colours used for each order in the plots.

**Additional Data 24.** Combined data of both the specimen details and shape measurements.
