# Supplementary figures and images for: Assessing the application of landmark-free morphometrics to macroevolutionary analyses
Source: BMC Ecol Evol. 2025 Apr 27;25:38. doi: 10.1186/s12862-025-02377-9 (PMC12034209; doi:10.1186/s12862-025-02377-9)

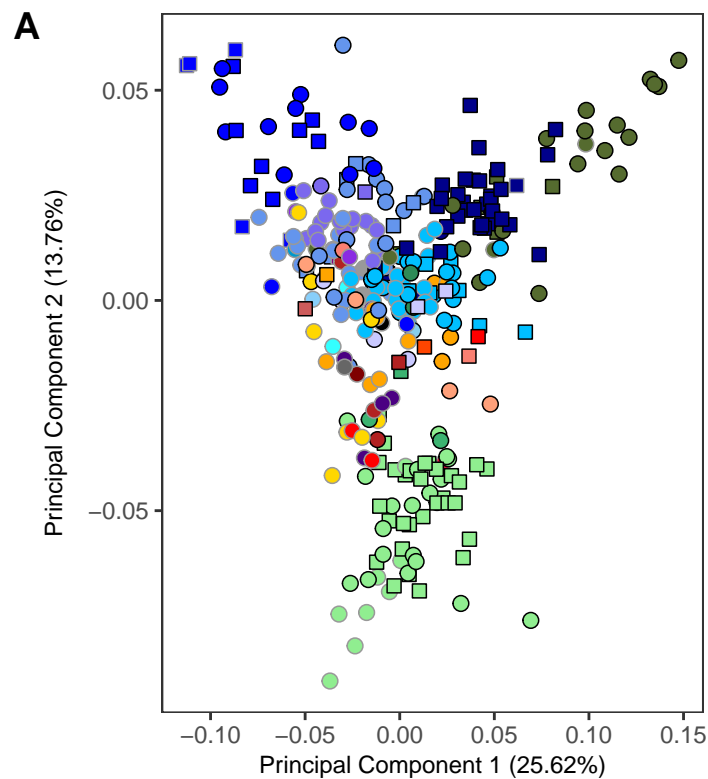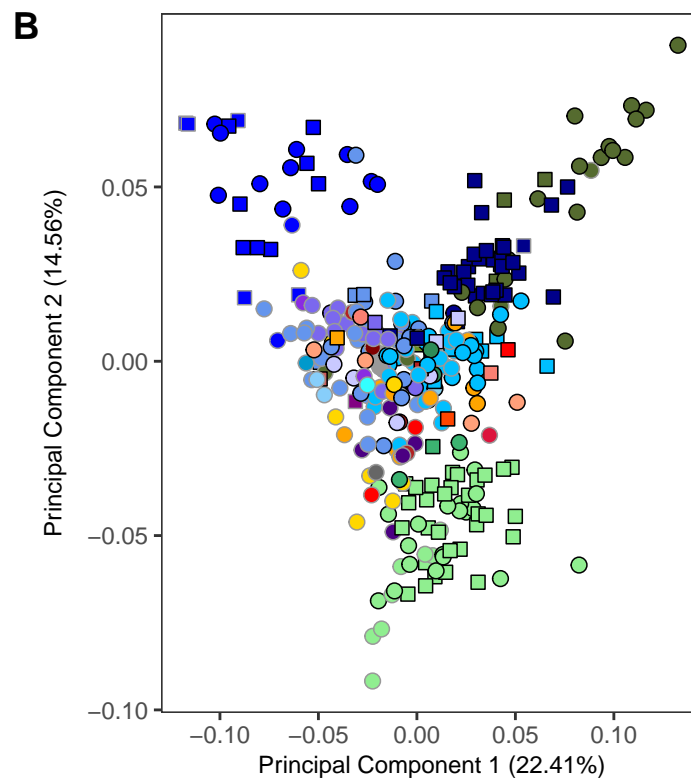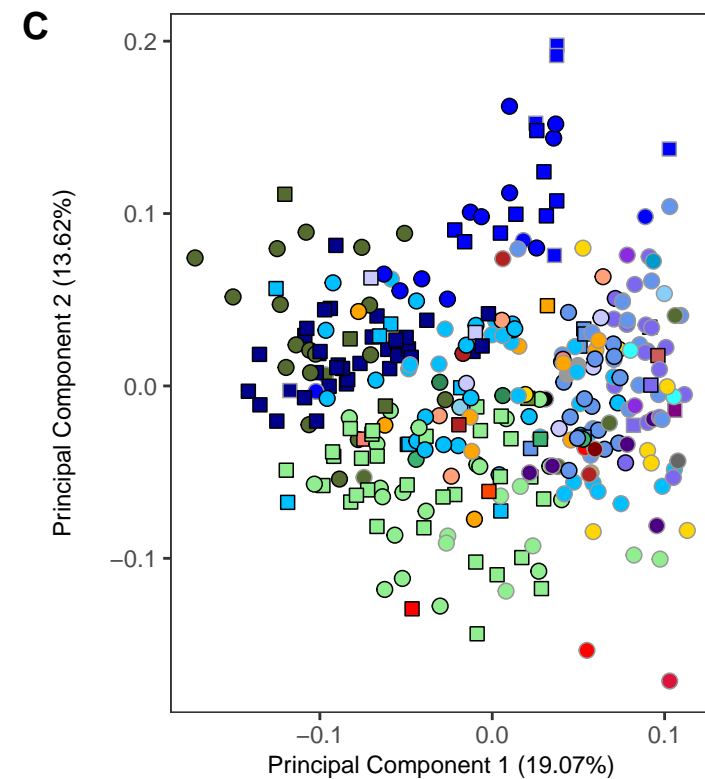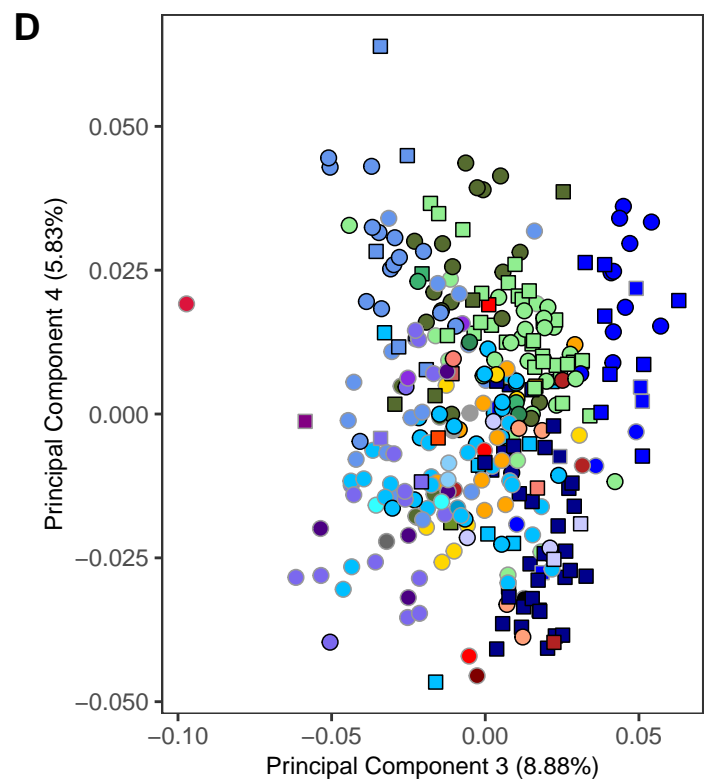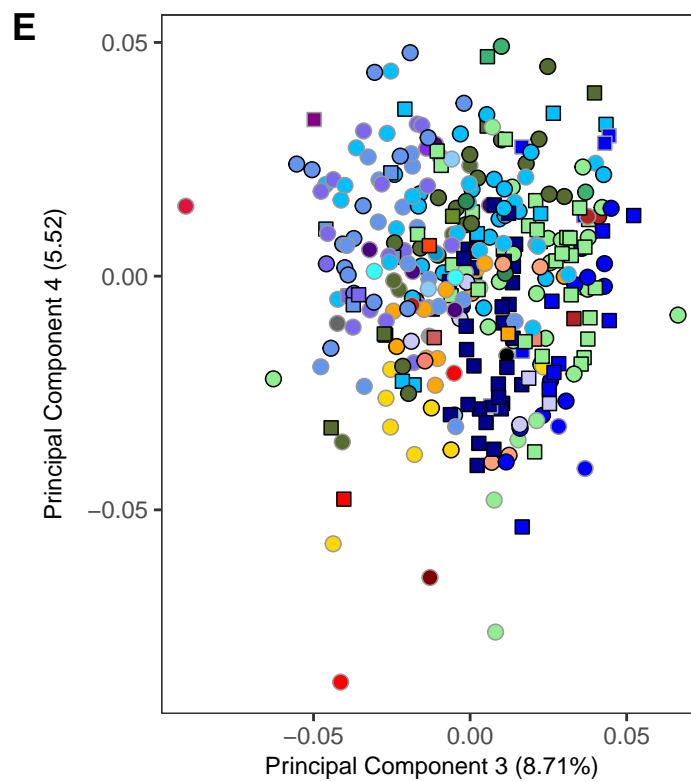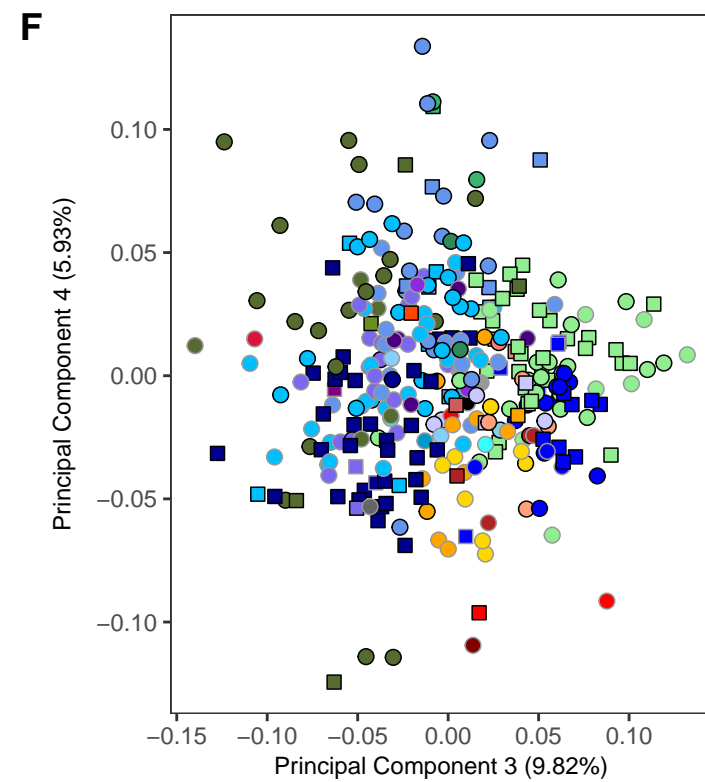

Supplement: Supplementary file 1 — Supplementary Material 1. [file 12862_2025_2377_MOESM1_ESM.zip › Supplementary Material/Figure A1/Figure A1 - Atlas Comparison.pdf]

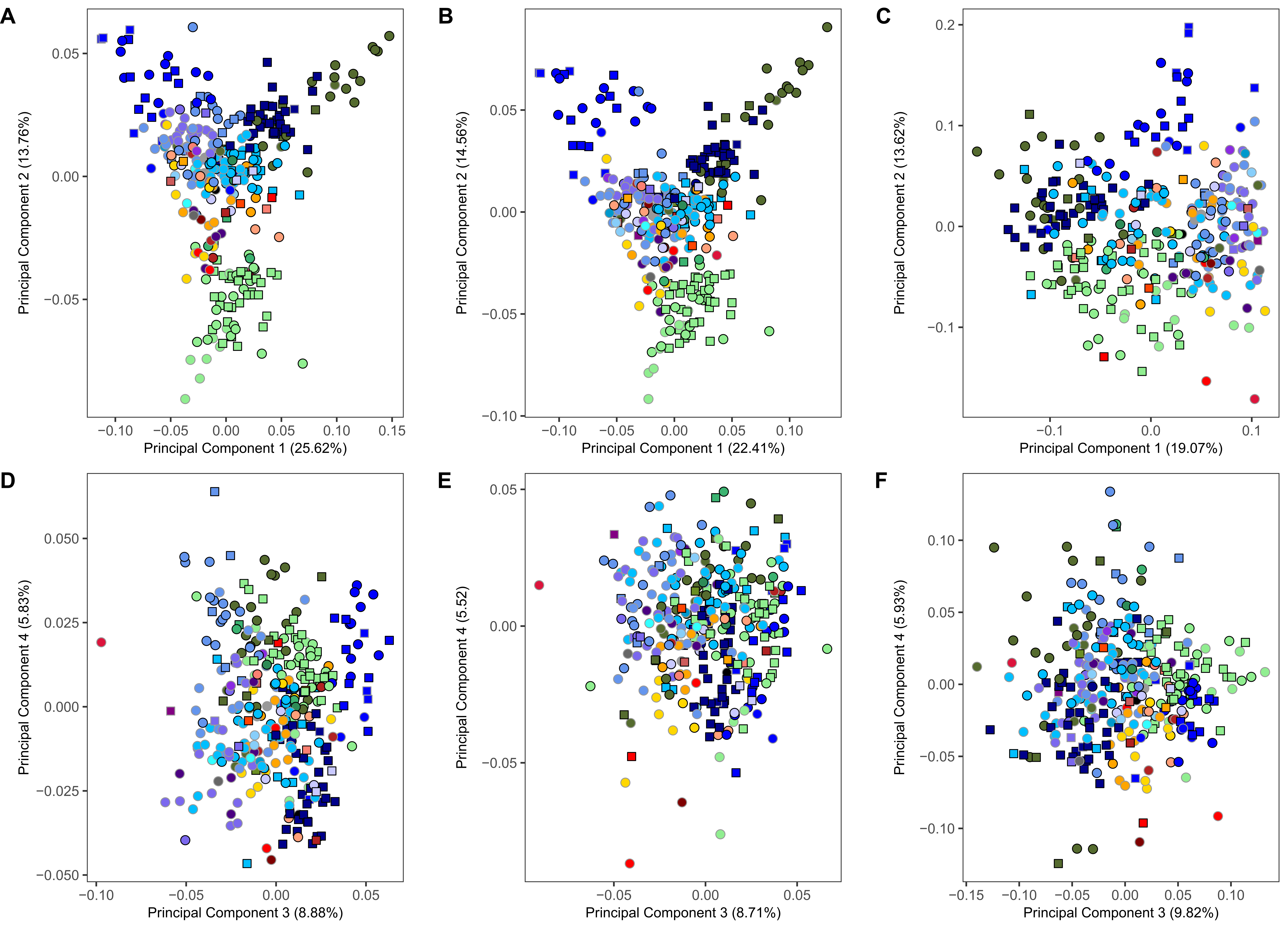

Supplement: Supplementary file 1 — Supplementary Material 1. [file 12862_2025_2377_MOESM1_ESM.zip › Supplementary Material/Figure A1/Figure A1 - Atlas Comparison.png]

**A**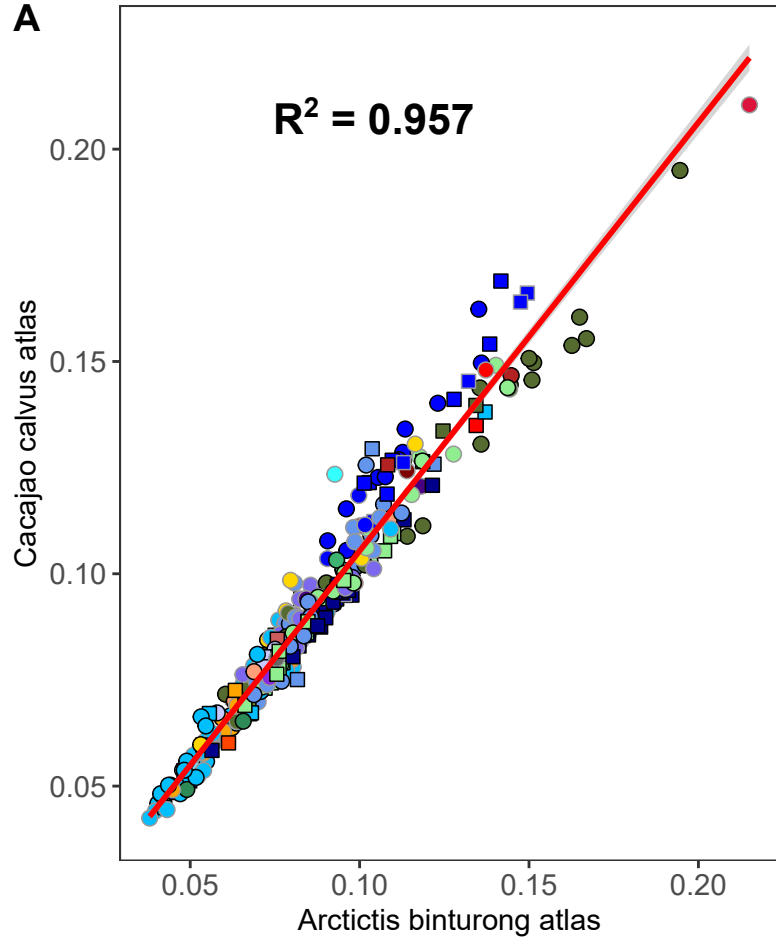**B**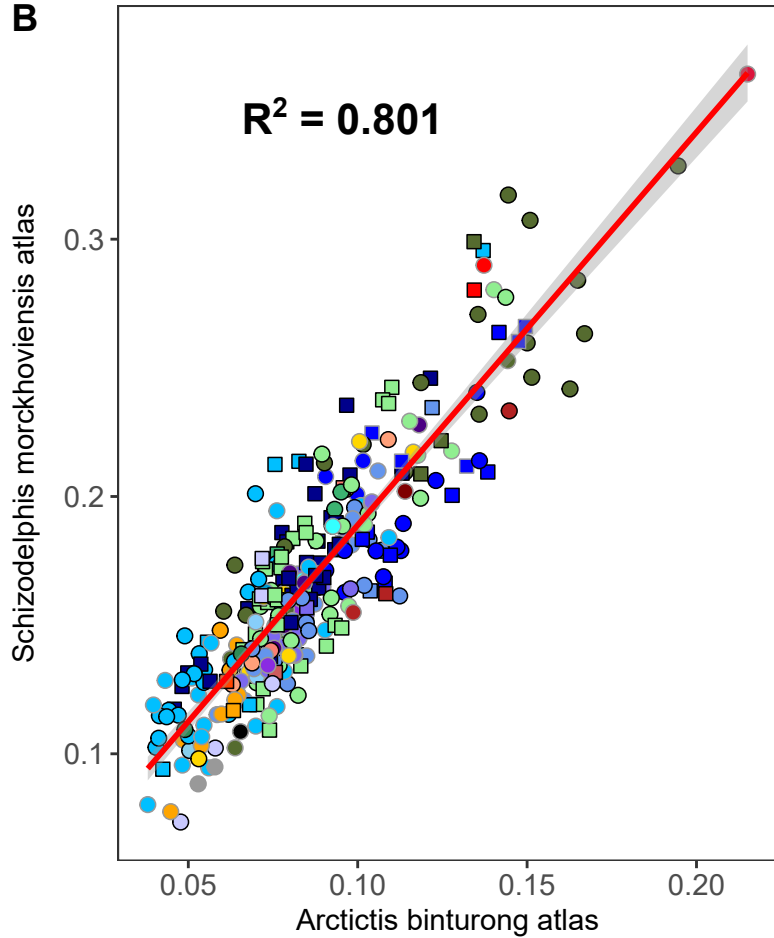

Supplement: Supplementary file 1 — Supplementary Material 1. [file 12862_2025_2377_MOESM1_ESM.zip › Supplementary Material/Figure A2/Figure A2 - Euclidean Atlas Comparison.pdf]

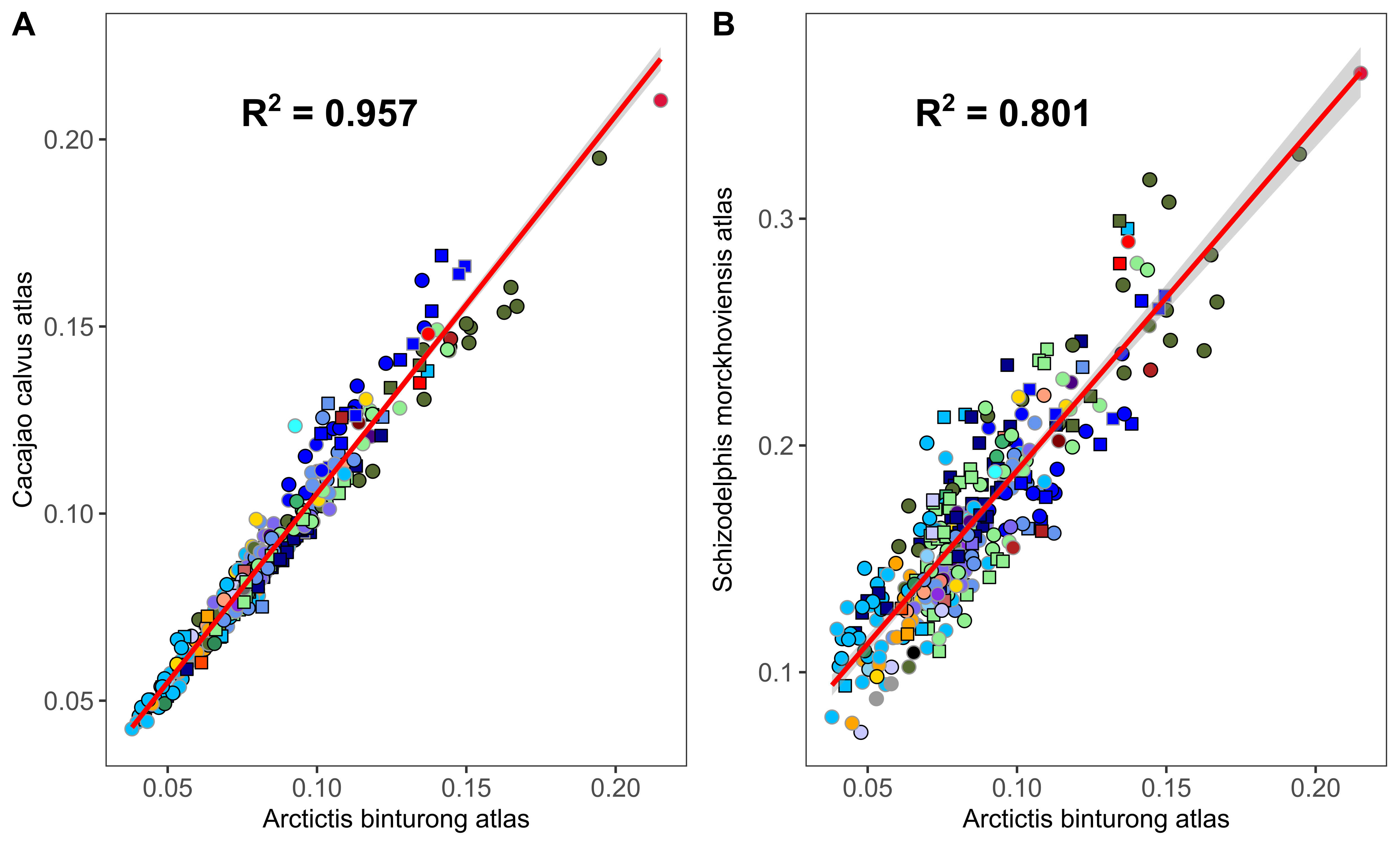

Supplement: Supplementary file 1 — Supplementary Material 1. [file 12862_2025_2377_MOESM1_ESM.zip › Supplementary Material/Figure A2/Figure A2 - Euclidean Atlas Comparison.png]

A

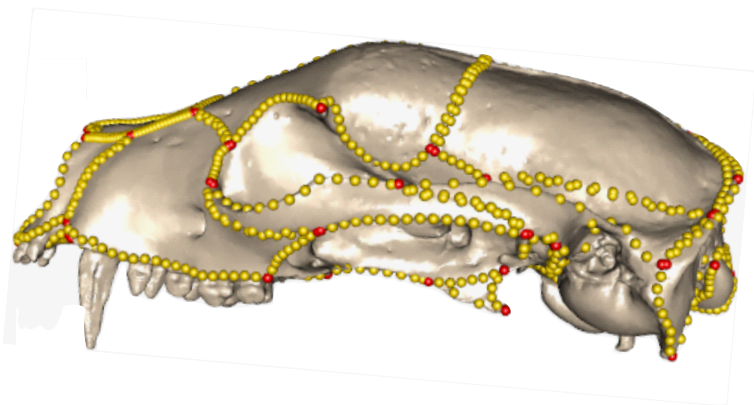

B

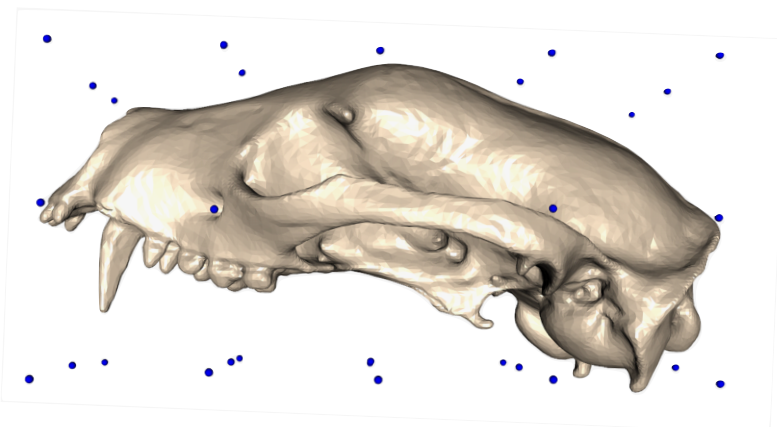

C

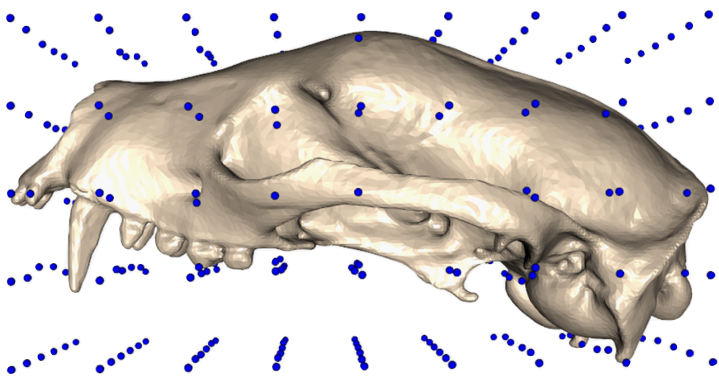

D

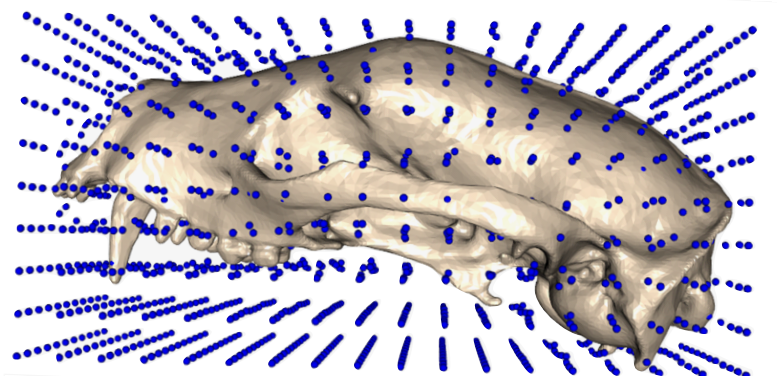

Supplement: Supplementary file 1 — Supplementary Material 1. [file 12862_2025_2377_MOESM1_ESM.zip › Supplementary Material/Figure A3/Figure A3- Comparison of manual landmarking scheme with a range of control points.pdf]

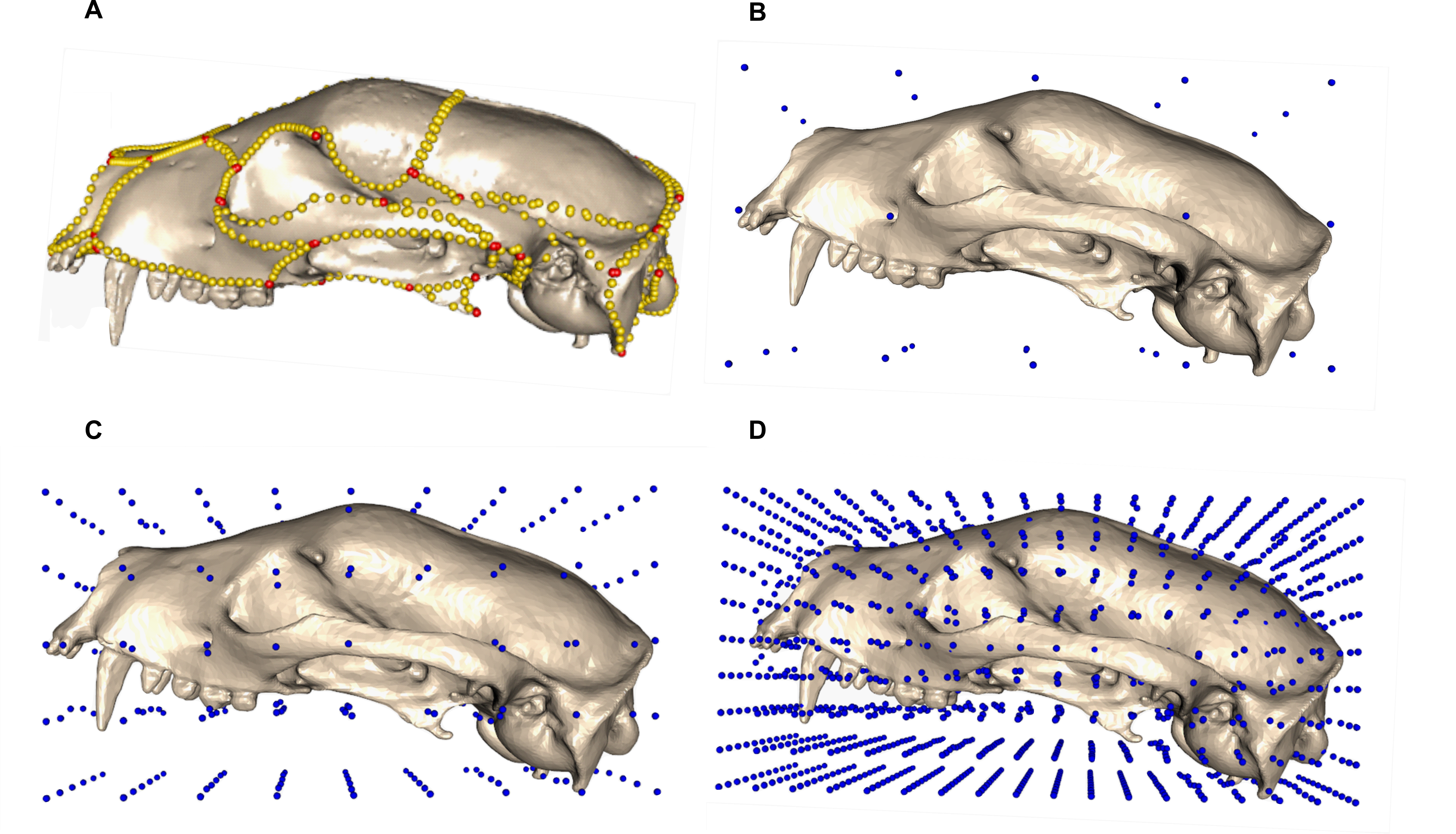

Supplement: Supplementary file 1 — Supplementary Material 1. [file 12862_2025_2377_MOESM1_ESM.zip › Supplementary Material/Figure A3/Figure A3- Comparison of manual landmarking scheme with a range of control points.png]

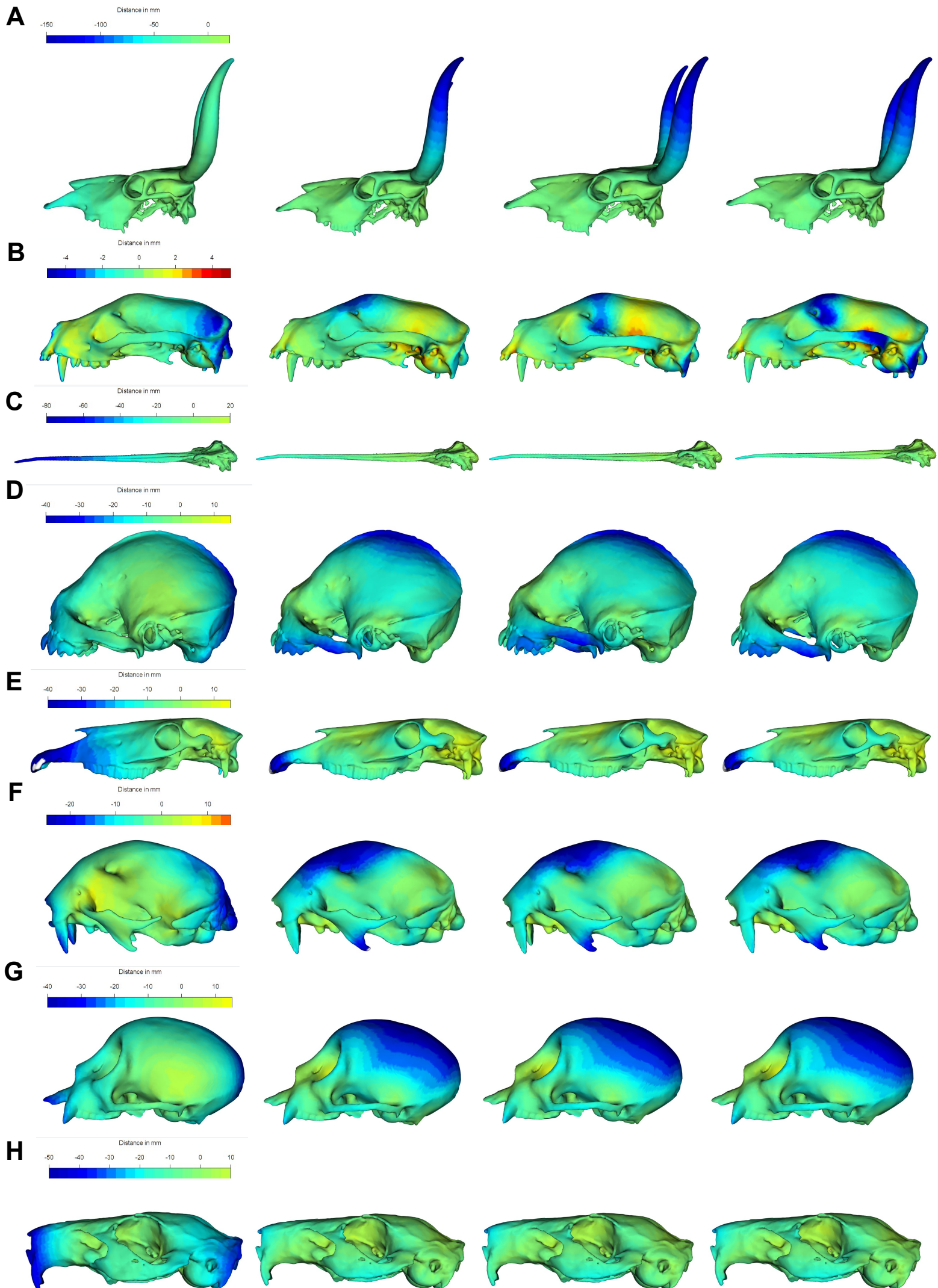

Supplement: Supplementary file 1 — Supplementary Material 1. [file 12862_2025_2377_MOESM1_ESM.zip › Supplementary Material/Figure A7/Figure A7 - Extended Heatmaps.pdf]

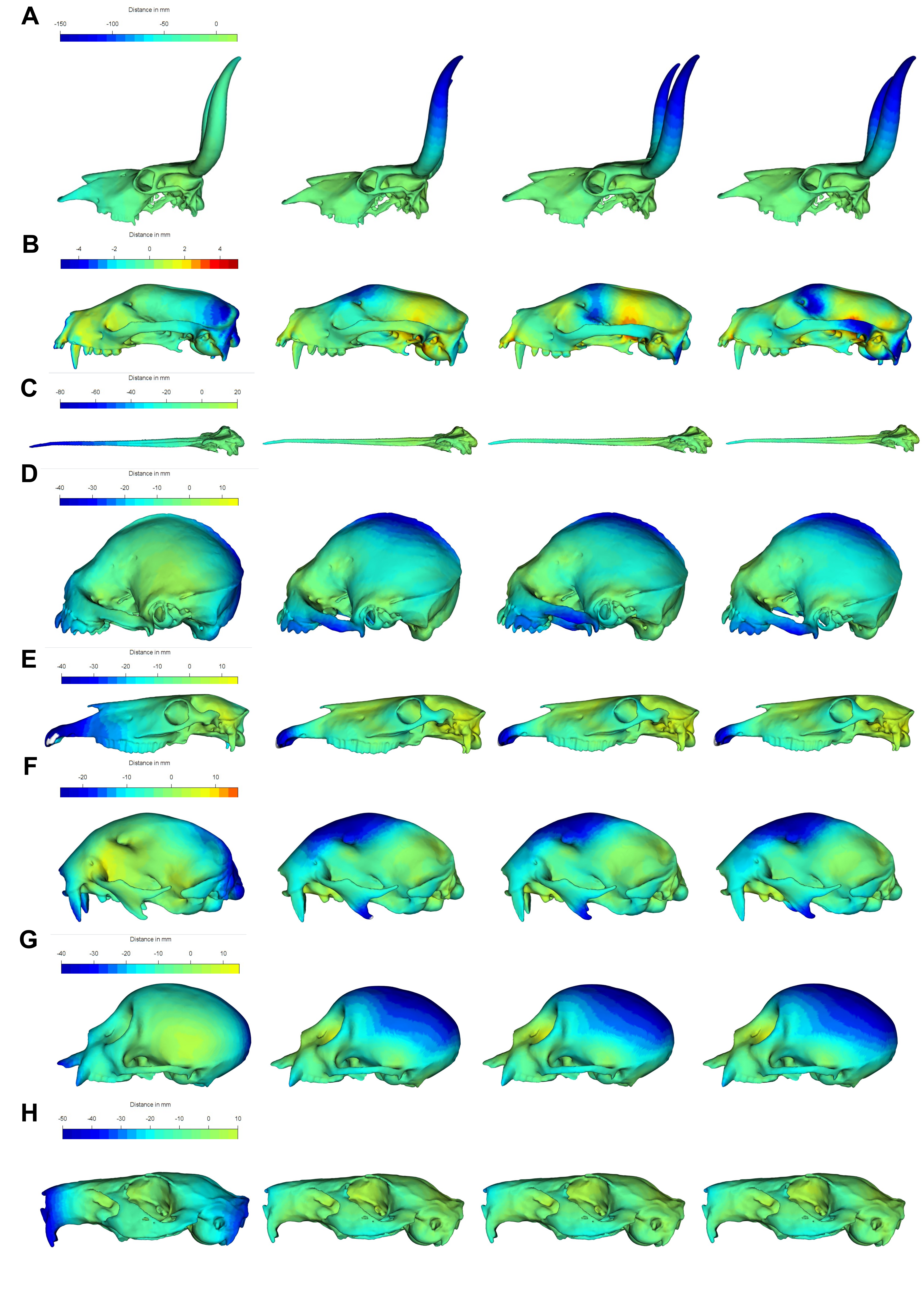

Supplement: Supplementary file 1 — Supplementary Material 1. [file 12862_2025_2377_MOESM1_ESM.zip › Supplementary Material/Figure A7/Figure A7 - Extended Heatmaps.png]

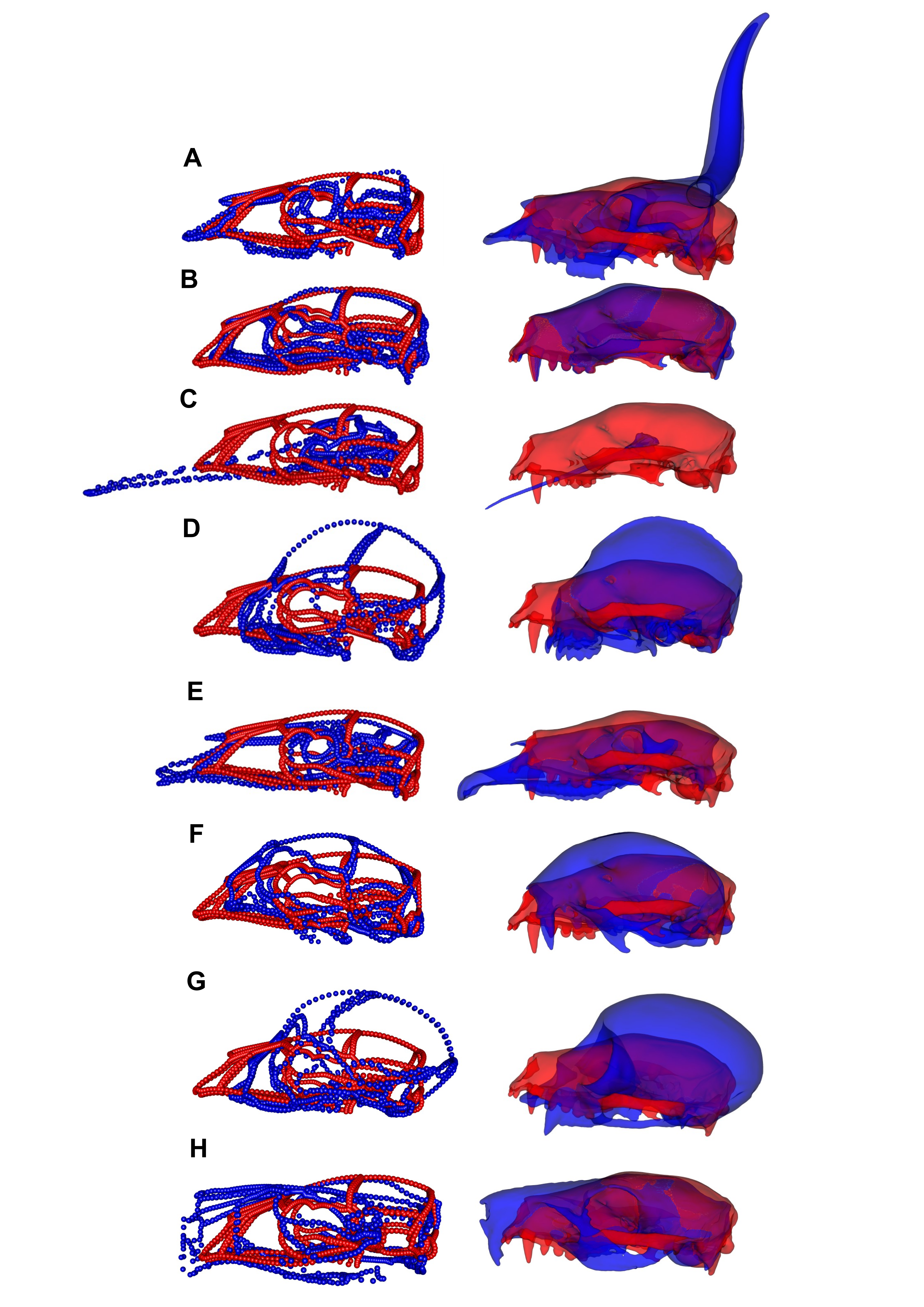

Supplement: Supplementary file 1 — Supplementary Material 1. [file 12862_2025_2377_MOESM1_ESM.zip › Supplementary Material/Figure A8/Figure A8 - Method_Alignment_Comparison.png]
