# Supplementary material for: Assessing the application of landmark-free morphometrics to macroevolutionary analyses
Source: BMC Ecol Evol. 2025 Apr 27;25:38. doi: 10.1186/s12862-025-02377-9 (PMC12034209; doi:10.1186/s12862-025-02377-9)
Supplement: Supplementary file 1 — Supplementary Material 1. [file Figure A8 - Method_Alignment_Comparison.pdf › Supplementary Material/Figure A8/Figure A8 - Method_Alignment_Comparison.pdf]

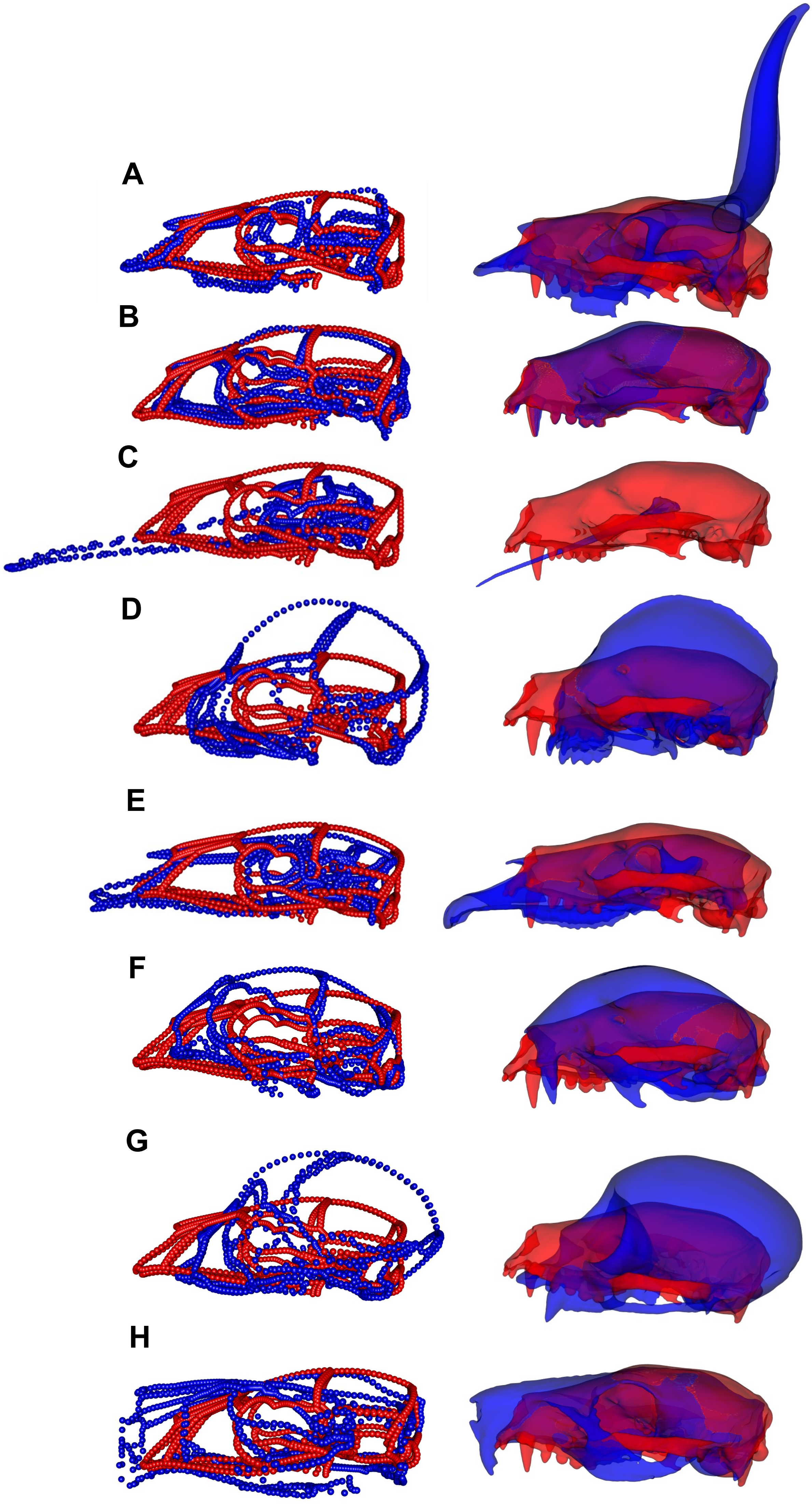
A
B
C
D
E
F
G
H
